# PDZD8-deficient mice accumulate cholesteryl esters in the brain as a result of impaired lipophagy

**DOI:** 10.1016/j.isci.2022.105612

**Published:** 2022-11-16

**Authors:** Keiko Morita, Mariko Wada, Kohta Nakatani, Yuki Matsumoto, Nahoki Hayashi, Ikuko Yamahata, Kotone Mitsunari, Nagi Mukae, Masatomo Takahashi, Yoshihiro Izumi, Takeshi Bamba, Michiko Shirane

**Affiliations:** 1 Department of Molecular Biology, Graduate School of Pharmaceutical Sciences, Nagoya City University, Nagoya, Aichi 467-8603, Japan; 2 Division of Metabolomics, Medical Institute of Bioregulation, Kyushu University, Fukuoka, Fukuoka 812-8582, Japan

**Keywords:** Biological sciences, Molecular biology, Neuroscience

## Abstract

Dyslipidemia including the accumulation of cholesteryl esters (CEs) in the brain is associated with neurological disorders, although the underlying mechanism has been unclear. PDZD8, a Rab7 effector protein, transfers lipids between endoplasmic reticulum (ER) and Rab7-positive organelles and thereby promotes endolysosome maturation and contributes to the maintenance of neuronal integrity. Here we show that CEs accumulate in the brain of PDZD8-deficient mice as a result of impaired lipophagy. This CE accumulation was not affected by diet, implicating a defect in intracellular lipid metabolism. Whereas cholesterol synthesis appeared normal, degradation of lipid droplets (LDs) was defective, in the brain of PDZD8-deficient mice. PDZD8 may mediate the exchange of cholesterol and phosphatidylserine between ER and Rab7-positive organelles to promote the fusion of CE-containing LDs with lysosomes for their degradation. Our results thus suggest that PDZD8 promotes clearance of CEs from the brain by lipophagy, with this role of PDZD8 likely contributing to brain function.

## Highlights

• PDZD8 mediates the exchange of PS and cholesterol between ER and Rab7^+^ organelles
• PDZD8 promotes the maturation and fusion of lysosomes with LDs, leading to lipophagy
• Deficiency of PDZD8 results in CE accumulation and consequent dyslipidemia in brain
• PDZD8 thus contributes to brain function through the clearance of CEs

## Graphical abstract

**Figure:**
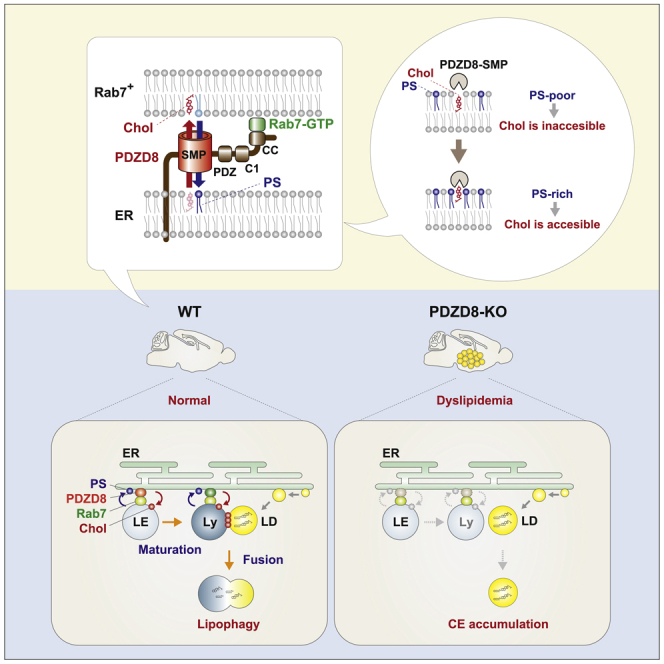

## Introduction

Dyslipidemia including cholesterol storage disease is associated with neurodegenerative disorders such as Alzheimer’s disease, Huntington’s disease, and Parkinson’s disease^1^^,^^2^^,^^3^^,^^4^^,^^5^ Intracellular cholesterol and its derivatives including cholesteryl esters (CEs) are either supplied by low-density lipoprotein (LDL) or synthesized in the endoplasmic reticulum (ER), and they are stored in lipid droplets (LDs). The abnormal accumulation of CEs has been attributed to the dysfunction of late endosomes and lysosomes (LEs/Lys).^6^ However, the detailed mechanism underlying dyslipidemia in the brain has remained unclear.

Membrane contact sites (MCSs) of intracellular organelles are regions where different organelles are closely associated, and they play an important role in the interorganelle communication. The main functions of MCSs include lipid transfer between organelles, calcium regulation, and control of organelle dynamics.^7^^,^^8^^,^^9^ In particular, MCSs between ER and LEs/Lys play a key role in mediating the intracellular distribution of cholesterol.^10^^,^^11^^,^^12^^,^^13^

PDZD8 forms a complex with Protrudin, VAP, and Rab7 at MCSs between ER and LEs/Lys. It mediates the transfer of lipids from ER to LEs/Lys and thereby promotes endosome maturation and maintains neuronal integrity.^14^^,^^15^^,^^16^^,^^17^^,^^18^^,^^19^^,^^20^^,^^21^ The components of this PDZD8 complex are associated with various neurological disorders,^15^^,^^22^^,^^23^^,^^24^^,^^25^^,^^26^ with mutation of PDZD8 itself being a risk factor for human intellectual disability including cognitive impairment^27^ and for posttraumatic stress disorder.^28^ However, the physiological function of PDZD8, especially in the brain, has not been fully elucidated.

Endosome maturation is a continuous process that includes the conversion of LEs to lysosomes^29^ as well as the fusion of lysosomes with substrate organelles.^12^^,^^30^^,^^31^^,^^32^ Cholesterol promotes membrane fusion between Rab7-positive organelles (endolysosomes) and autophagosomes.^33^ Furthermore, active Rab7 facilitates the incorporation of LDs into lysosomes by the process known as lipophagy.^34^ Rab7 and cholesterol have thus been implicated in the execution of lipophagy,^35^^,^^36^^,^^37^ but the key molecules involved in the fusion of LDs with and their degradation by endolysosomes have been unclear.

We have now found that PDZD8 knockout (KO) mice manifest abnormal CE accumulation in the brain that results from an impairment of lipophagy. This impairment is itself due to defective lysosomal maturation dependent on Rab7 and cholesterol. Our results thus suggest that PDZD8 plays an essential role in maintaining the integrity of brain function by regulating cholesterol metabolism.

## Results

### Abnormal cholesteryl ester accumulation in the brain of PDZD8-deficient mice

**Figure 1:**
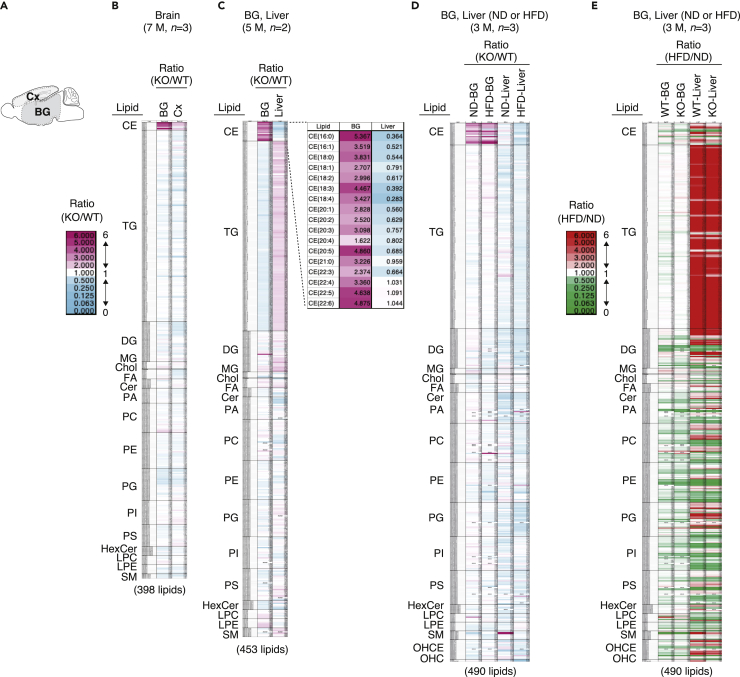
Abnormal CE accumulation in the brain of PDZD8-deficient mice (A) Schematic representation of mouse brain regions subjected to lipidome analysis. (B–D) Heat maps of lipid amount ratios for PDZD8-KO relative to WT mice as determined by lipidome analysis. The ratios are shown according to the indicated color scale (B, left). The tissues compared in each analysis were obtained from the same mice, but the mice fed the ND or the HFD in (D) were different. (B) BG and Cx for three mice at 7 months (M) of age. (C) BG and liver from two mice at 5 months of age, with the ratios for each type of CE being shown to the right of the heat map. (D) BG and liver for three ND- or HFD-fed mice at 3 months of age (HFD feeding for 1 month). (E) Heat maps of lipid amount ratios for HFD-fed mice relative to ND-fed mice determined by lipidome analysis. The ratios are shown according to the indicated color scale (left) and were determined for the mice analyzed in (D). Lipid abbreviations: CE, cholesteryl ester; TG, triacylglycerol; DG, diacylglycerol; MG, monoacylglycerol; Chol, cholesterol; FA, fatty acid; Cer, ceramide; PA, phosphatidic acid; PC, phosphatidylcholine; PE, phosphatidylethanolamine; PG, phosphatidylglycerol; PI, phosphatidylinositol; PS, phosphatidylserine; HexCer, hexosylceramide; LPC, lyso-PC; LPE, lyso-PE; SM, sphingomyelin; OHCE, oxidized CE; OHC, oxysterol. See also Figures S1 and S2.

Lipidosis or abnormal lipid accumulation in the brain is associated with neurological disorders. Given that PDZD8 possesses lipid transfer activity that promotes endosome maturation and thereby maintains neuronal integrity, we investigated lipid abnormalities in the brain of PDZD8-KO mice by lipidome analysis. For this analysis, we applied supercritical fluid chromatography-triple-quadrupole mass spectrometry (SFC/QqQMS), a novel method for quantitative lipid analysis that allows the comprehensive measurement of lipid molecules with high sensitivity. The basal ganglia (BG) and cortex (Cx) of the brain were sampled (Figure 1A). PDZD8-KO mice showed a marked abnormal accumulation specifically of CEs in the BG, with this effect being less pronounced in the Cx, compared with wild-type (WT) mice (Figure 1B). Comparison of brain versus liver revealed abnormal CE accumulation in the BG but not in the liver of PDZD8-KO (Figures 1C and 1D).

To investigate whether this abnormal CE accumulation in the brain of PDZD8-KO mice was affected by diet, we compared the lipidomes of mice fed either a normal diet (ND) or a high-fat diet (HFD). The KO/WT ratio for CE content showed a similar increase for the BG, but not for the liver, of mice fed either diet (Figure 1D). Whereas both WT and PDZD8-KO mice showed marked increases in both CE and triglyceride (TG) content of the liver in response to feeding with the HFD, no such changes were apparent for the BG (Figure 1E). The CE content of the brain thus appeared to be largely unaffected by diet.

### Diet-independent accumulation of cholesteryl esters in the brain of PDZD8-KO mice

**Figure 2:**
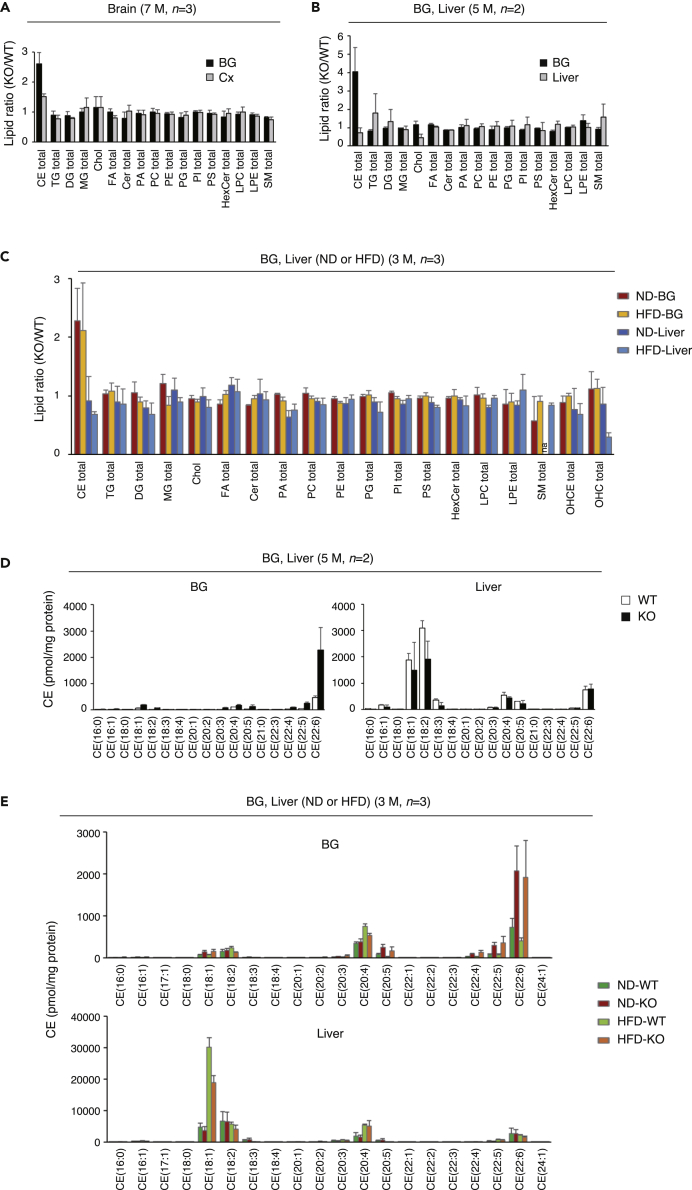
Diet-independent dyslipidemia in the brain of PDZD8-deficient mice (A–C) Lipid amount ratios (PDZD8-KO/WT) for lipid classes in the BG and Cx as in Figure 1B, in the BG and liver as in Figure 1C, or in the BG and liver of ND- or HFD-fed mice as in Figure 1D, respectively. The ratios were determined as the mean + SD in each case. (D and E) Amount of each type of CE in the BG and liver of WT and PDZD8-KO mice as in Figure 1C or in Figure 1D, respectively. Data (pmol/mg protein) are the mean + SD in each case. See also Figures S1 and S2.

**Figure 3:**
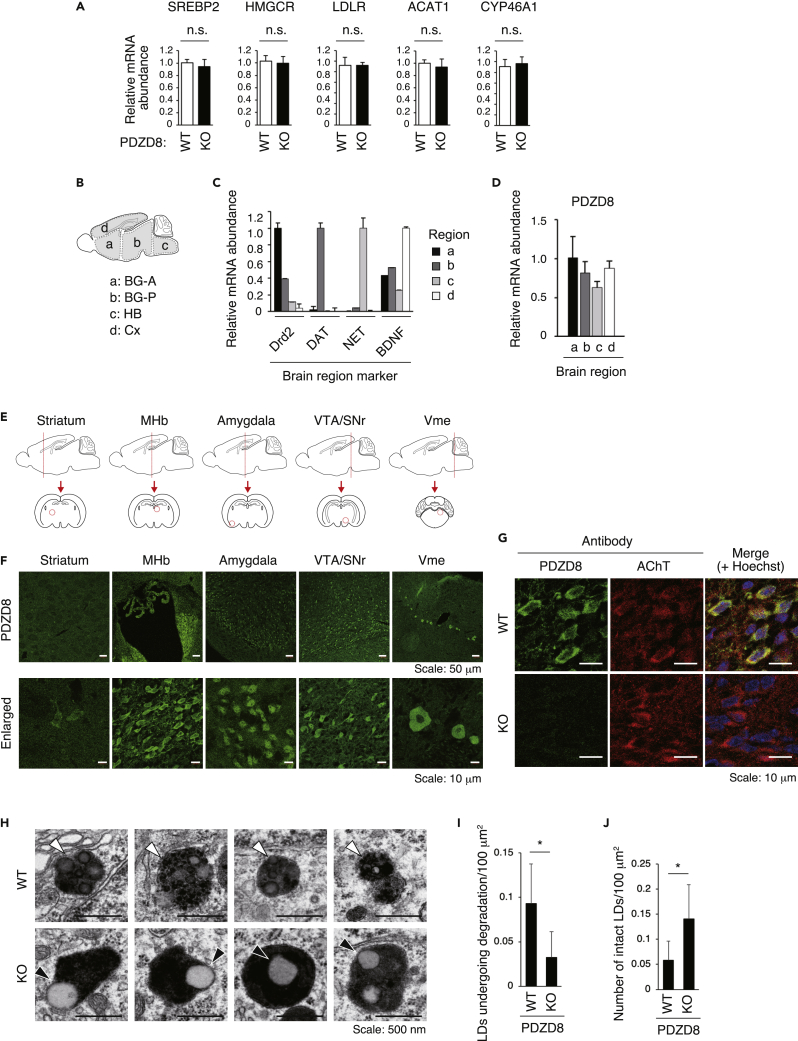
Defective lipophagy in the brain of PDZD8-KO mice (A) Relative mRNA abundance for the cholesterol-related proteins SREBP2, HMGCR, LDLR, ACAT1, and CYP46A1 in the BG of WT or PDZD8-KO mice at 5 months of age (*n* = 2). Data are means + SD. Differences between genotypes were not significant (n.s.) by Student’s t test. (B) Schematic representation of mouse brain regions subjected to RT and real-time PCR analysis in (C) and (D). A, anterior; P, posterior; HB, hindbrain. (C) Relative mRNA abundance for dopamine receptor D2 (Drd2), dopamine transporter (DAT), norepinephrine transporter (NET), and brain-derived neurotrophic factor (BDNF) as markers for brain regions a to d indicated in (B) from 3-month-old WT mice. Data are means +SD (*n* = 2). (D) Relative mRNA abundance for PDZD8 in brain regions a to d from 3-month-old WT mice. Data are means +SD (*n* = 2). (E) Schematic representation of sectioning of the mouse brain for the examination of the striatum, medial habenula (MHb), amygdala, ventral tegmental area (VTA)/substantia nigra pars reticulata (SNr), and trigeminal mesencephalic nucleus (Vme). The red lines in the sagittal sections (upper) indicate the position of the coronal sections (lower), in which the red circles indicate the regions corresponding to the images in (F). (F) Immunofluorescence analysis of PDZD8 (green) in the WT brain. Enlarged images are also shown (lower). (G) Immunofluorescence analysis of PDZD8 (green) and AChT (red) for MHb neurons in the WT or PDZD8-KO brain. Nuclear staining with Hoechst 33342 (blue) is also shown in the merged images. (H) TEM images of lipophagy in MHb neurons of the WT or PDZD8-KO brain. White arrowheads indicate LDs fused with lysosomes and undergoing degradation. Black arrowheads indicate LDs making contact but not fusing with lysosomes and not undergoing degradation. (I and J) Number of LDs undergoing degradation per 100 μm^2^ (I) or number of intact LDs per 100 μm^2^ (J) as shown in (H). Data are means +SD (*n* = 26 and 28 images for WT and PDZD8-KO mice, respectively). ∗p < 0.05 (Student’s t test). See also Figure S3.

The lipidome data for the brain and liver (Figures 1B–1E) were then analyzed in more detail. The total amount of CEs in the BG of PDZD8-KO mice showed an ∼3-fold increase relative to WT mice at 7 months of age (Figure 2A). Similar increases of ∼4-fold and ∼2-fold were apparent for the BG of the mutant mice at 5 months (Figure 2B) and 3 months (Figure 2C) of age, respectively, whereas no differences were apparent for the liver between the two genotypes (Figures 2B and 2C). The increase in CE content in the BG of PDZD8-KO mice relative to WT mice was not affected by feeding with the HFD compared with the ND (Figure 2C). Together, these results revealed the abnormal accumulation of CEs in the brain, in particular in the BG, of PDZD8-KO mice at all ages examined (3 to 7 months).

We next investigated why only CEs out of all lipids accumulated in the brain of PDZD8-KO mice and why such an effect was apparent in the brain but not in the liver. Reverse transcription (RT) and real-time polymerase chain reaction (PCR) analysis revealed that the abundance of PDZD8 mRNA was much higher in the brain than in the liver of WT mice (Figure S1A), suggesting that PDZD8 function with regard to CE metabolism may be more important in the brain. We also found that the amount of CEs was markedly lower in the brain than in the liver, whereas the abundance of other lipids was higher in the brain or similar between the two organs (Figure S1B), suggesting that the CE content of the brain might need to be maintained low by constitutive regulation. Classification of CEs according to the type of fatty acid (FA) constituent revealed that the amounts of all types of CE in the BG were higher for PDZD8-KO mice than for WT mice at 5 months of age, with CE(22:6) showing the largest absolute increase (Figures 2D and S2A). No such large difference in the amount of any type of CE was apparent for the liver of PDZD8-KO versus WT mice (Figure 2D). All types of CE were also more abundant in the BG of PDZD8-KO mice than in the BG of WT mice at 7 months of age, with CE(22:6) again showing the largest absolute increase (Figures S2B and S2C). The most abundant types of CE in WT differed between the BG and the liver, with CE(22:6) and CE(20:4) being most abundant in the BG and CE(18:1) and CE(18:2) being most abundant in the liver (Figure 2E). In the liver, unlike BG, the amount of CE(18:1) showed a large increase in response to HFD feeding compared with ND feeding, with no substantial difference being apparent between WT and PDZD8-KO animals (Figure 2E). Whereas lipid content in the liver is well known to be affected by diet, the insensitivity of that in the brain to food type has not previously been described. Our results thus suggested that the abnormal CE accumulation in the brain of PDZD8-KO mice is not dependent on cellular uptake of diet-derived LDL, but rather is related to either excessive synthesis or impaired metabolism of CE-containing LDs within cells.

### Defective lipophagy in the brain of PDZD8-KO mice

We then examined whether PDZD8 might play a role in cholesterol synthesis. Cholesterol is synthesized and modified in ER, with cholesterol-related gene expression in the nucleus being regulated by a feedback system (Figure S3). Hydroxymethylglutaryl-CoA reductase (HMGCR) catalyzes the conversion of HMG-CoA to mevalonate in the cholesterol biosynthetic pathway, and cholesterol is subsequently converted to derivatives such as CEs and oxysterol (24-OHC) by acyl-CoA:cholesterol acyltransferase 1 (ACAT1) and cholesterol 24-hydroxylase (CYP46A1), respectively. Intracellular cholesterol inhibits the expression of the gene for SREBP2, a transcription factor that regulates expression of genes including those for HMGCR and the LDL receptor (LDLR). However, the abundance of mRNAs for SREBP2, HMGCR, LDLR, ACAT1, and CYP46A1 in the BG did not differ between PDZD8-KO and WT mice (Figure 3A).

Next, in order to examine the degradation of CE-containing LDs in the brain, we attempted to identify brain regions in which PDZD8 is highly expressed. The brain of WT mice was divided into the following regions: the anterior portion of the BG (a), the posterior portion of the BG (b), the hindbrain (c), and the Cx (d) (Figure 3B). RT and real-time PCR analysis of regional marker gene expression confirmed the appropriate dissection of these brain regions (Figure 3C). Similar analysis of the same samples revealed that the abundance of PDZD8 mRNA was similar in all four brain regions (Figure 3D), suggesting that the accumulation of CEs to a greater extent in the BG than in the Cx of PDZD8-KO mice was not due simply to the expression level of PDZD8, with the presence or absence of a functionally complementary molecule possibly playing a role.

We also examined the regional expression of PDZD8 in the brain by immunohistofluorescence staining. This analysis detected PDZD8 in the striatum, medial habenula (MHb), amygdala, ventral tegmental area (VTA)/substantia nigra pars reticulata (SNr), and trigeminal mesencephalic nucleus (Vme) (Figures 3E and 3F). Among these regions, despite its small size, the MHb was the most suitable for examination by transmission electron microscopy (TEM), given that PDZD8 was highly expressed in almost all its component cells (Figure 3F). The specificity of the PDZD8 antibody signal in the MHb was confirmed by its loss in PDZD8-KO mice, and the PDZD8-expressing cells were positive for acetylcholine transferase (AChT) (Figure 3G). We then examined lysosomes and LDs, or lipophagy, in MHb neurons by TEM. LDs (which appeared white) seemed to make contact or to undergo fusion with lysosomes (which appeared black) in PDZD8-KO and WT neurons, respectively (Figure 3H). Whereas most LDs in WT neurons appeared to undergo degradation, as suggested by an overall gray coloration, segmentation, and indistinct boundaries with lysosomes, those in PDZD8-KO neurons seemed not to undergo degradation, remaining distinct and intact (Figures 3H–3J). These results suggested that the abnormal accumulation of CEs in the brain of PDZD8-KO mice may result from a failure of LD degradation due to insufficient lipophagy.

### PDZD8 possesses phosphatidylserine and cholesterol transfer activity

**Figure 4:**
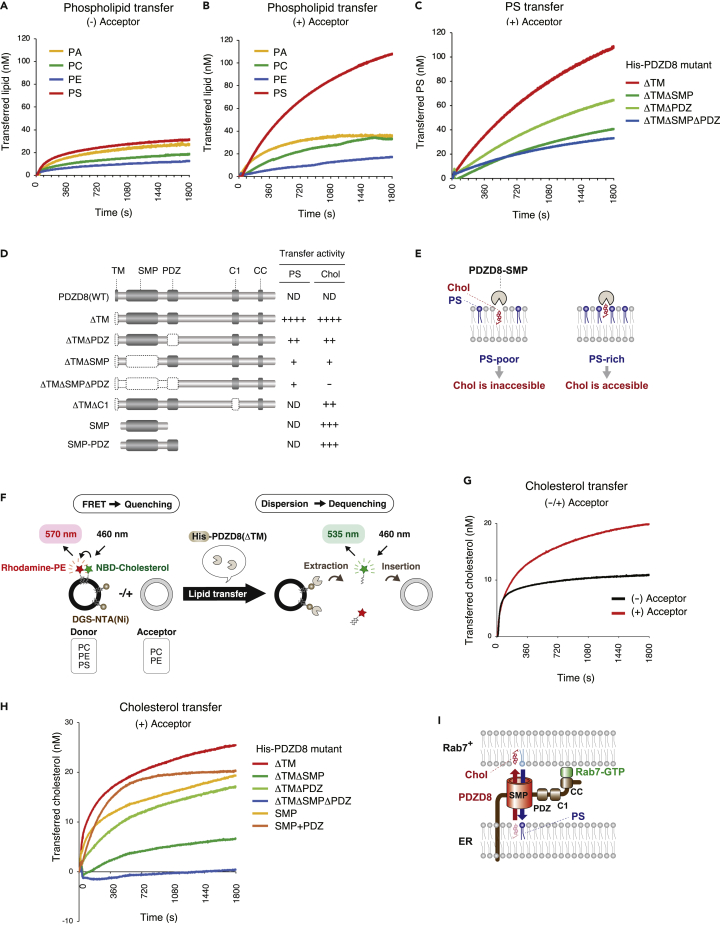
PDZD8 possesses PS and cholesterol transfer activity (A and B) Phospholipid transfer activity of His_6_-PDZD8(ΔTM) in the absence (A) or presence (B) of acceptor liposomes as determined with the liposome-FRET assay shown in Figure S4A. The amount of transferred lipid (nM) is shown. (C) PS transfer activity of His_6_-PDZD8 deletion mutants in the presence of acceptor liposomes. (D) Domain structure of mouse wild-type PDZD8 [PDZD8(WT)] and its deletion mutants. A summary of the PS and cholesterol transfer activities of each mutant determined as in (C) and (H), respectively, is shown on the right. ND, not determined. (E) Schematic representation of model for the accessibility of cholesterol to PDZD8-SMP in PS-poor (left) or PS-rich (right) domains of a lipid bilayer. (F) Schematic representation of the liposome-FRET assay for cholesterol transfer by His_6_-PDZD8(ΔTM) as performed with donor liposomes containing rhodamine-PE, NBD-cholesterol, and DGS-NTA(Ni) and in the absence or presence of acceptor liposomes. The lipid constituents of the liposomes are shown in the boxes later in discussion. (G) Cholesterol transfer activity of PDZD8(ΔTM) in the absence or presence of acceptor liposomes. (H) Cholesterol transfer activity of the indicated PDZD8 deletion mutants in the presence of acceptor liposomes. (I) Model for the mechanism of lipid transfer by PDZD8, indicating that PDZD8 exchanges PS and cholesterol between the ER and Rab7-positive organelles in a manner dependent on its SMP domain. See also Figure S4.

We previously showed that PDZD8 possesses lipid extraction activity with donor liposomes but not lipid insertion activity with acceptor liposomes.^16^ We here modified our *in vitro* fluorescence resonance energy transfer (FRET)-based liposome assay to examine further this activity of PDZD8. In the new version of the assay, PDZD8 was anchored to the donor membrane in order to increase the efficiency of lipid extraction by including in the donor liposomes DGS-NTA(Ni), a lipid conjugated to nitrilotriacetic acid (Ni^2+^ salt) that allows the docking of hexahistidine (His_6_)-tagged recombinant proteins,^38^ and the measurement time was extended from 300 to 1800 s (Figure S4A). In this assay, donor liposomes containing rhodamine-labeled phosphatidylethanolamine (PE) and nitrobenzoxadiazole (NBD)-labeled phospholipid give rise to FRET that is abrogated by lipid transfer (Figure S4A). NBD fluorescence would be expected to saturate rapidly if the test protein mediates only lipid extraction, whereas it would be expected to continue to increase if the protein mediates lipid transfer (including both lipid extraction and insertion). Although phosphatidic acid (PA), phosphatidylcholine (PC), and phosphatidylethanolamine (PE) were only extracted from donor liposomes, phosphatidylserine (PS) was both extracted from and inserted into liposomes by His_6_-tagged PDZD8(ΔTM), a mutant form of PDZD8 lacking the transmembrane domain (Figures 4A, 4B, and S4B). In this setting, however, it would be possible for PS to be transferred in both directions, given that the donor and acceptor liposomes have the same base lipid composition, including PC and PE (Figure S4A). The PS transfer activity of various His_6_-tagged forms of PDZD8 was calculated by subtracting the assay value obtained with His_6_-tagged glutathione S-transferase (GST) as a negative control from that obtained with each PDZD8 mutant, and the activity of PDZD8(ΔTM) was found to be markedly reduced by the deletion of the SMP domain (Figures 4C and 4D).

Cholesterol-binding proteins, such as GRAMD1s, are able to access cholesterol in a PS-rich membrane domain.^39^ Thus, we predicted that the SMP domain of PDZD8 might also be more accessible to cholesterol in the PS-rich membrane domain (Figure 4E). We therefore next investigated the potential cholesterol transfer activity of PDZD8 by further modifying the liposome-FRET assay to include PS in the donor liposomes (Figure 4F). Comparison of fluorescence values obtained with His_6_-PDZD8(ΔTM) in the absence or presence of acceptor liposomes revealed that the fluorescence saturated rapidly in the former instance and continued to increase in the latter, suggesting that PDZD8 possesses both cholesterol extraction and cholesterol insertion activity—that is, cholesterol transfer activity (Figure 4G). Cholesterol transfer is unidirectional in this system as the base lipids of the donor and acceptor liposomes have different compositions (Figure 4F). Examination of the cholesterol transfer activity of various His_6_-tagged PDZD8 mutants revealed that such activity was markedly reduced by the deletion of the SMP domain and completely lost after the deletion of both the SMP and PDZ domains (Figures 4D and 4H). In contrast, SMP and SMP + PDZ fragments showed high activity, almost equal to that of PDZD8(ΔTM). These results thus implicated the SMP domain as the region responsible for the cholesterol transfer activity of PDZD8, with the PDZ domain playing a supporting role.

PDZD8 is an ER-resident protein and its C1 domain binds to phosphatidylinositol 4-phosphate [PI(4)P],^16^ which is abundant in the endosome membrane.^40^ We, therefore, examined the possible role of the C1 domain and PI(4)P in cholesterol transfer by PDZD8. The activity of PDZD8(ΔTM) was markedly increased by the addition of PI(4)P to acceptor liposomes, whereas it was attenuated by the deletion of C1 (Figure S4C), suggesting that the association of the C1 domain of PDZD8 in the ER with PI(4)P in endosomes facilitates cholesterol transfer by PDZD8.

These findings and previous observations^16^^,^^17^^,^^18^^,^^19^ provide the basis for a model of the mechanism underlying PS and cholesterol transfer by PDZD8 (Figure 4I). The ER-resident protein PDZD8 thus tethers ER to Rab7-positive organelles through the binding of its CC domain to Rab7. PDZD8 may then transfer PS from Rab7-positive organelles to ER as well as cholesterol from PS-rich domains of the ER membrane to the Rab7-positive organelles in a manner dependent on its SMP domain (Figure 4I).

### PDZD8 regulates the subcellular localization of phosphatidylserine and cholesterol

**Figure 5:**
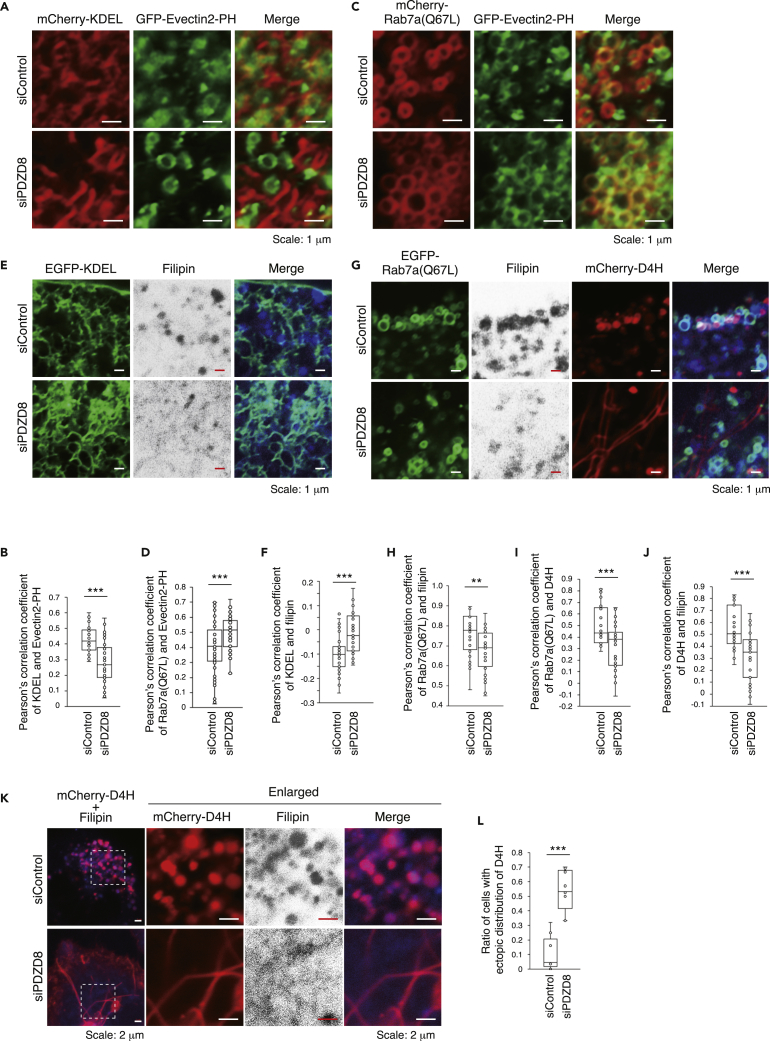
PDZD8 facilitates the localization of cholesterol to Rab7-positive organelles as well as that of PS to ER (A) Confocal fluorescence images of HeLa cells transfected with control (siControl) or PDZD8 (siPDZD8) siRNAs as well as with expression vectors for mCherry-KDEL (red) and GFP-Evectin2-PH (green). (B) Pearson’s correlation coefficient for the colocalization of mCherry-KDEL and GFP-Evectin2-PH in images similar to those in (A) (*n* = 28 and 41 cells for siControl and siPDZD8, respectively). (C) Confocal fluorescence images of HeLa cells transfected with siControl or siPDZD8 as well as with expression vectors for mCherry-Rab7a(Q67L) (red) and GFP-Evectin2-PH (green). (D) Pearson’s correlation coefficient for the colocalization of mCherry-Rab7a(Q67L) and GFP-Evectin2-PH in images similar to those in (C) (*n* = 57 and 50 cells for siControl and siPDZD8, respectively). (E) Confocal fluorescence images of HeLa cells transfected with siControl or siPDZD8 as well as with an expression vector for EGFP-KDEL (green) and then metabolically labeled with filipin (shown blue in the merged images). (F) Pearson’s correlation coefficient for colocalization of EGFP-KDEL and filipin in images similar to those in (E) (*n* = 36 and 35 cells for siControl and siPDZD8, respectively). (G) Confocal fluorescence images of HeLa cells transfected with siControl or siPDZD8 as well as with expression vectors for EGFP-Rab7a(Q67L) (green) and mCherry-D4H (red) and then metabolically labeled with filipin (shown blue in the merged images). (H–J) Pearson’s correlation coefficient for the colocalization of EGFP-Rab7a(Q67L) and filipin (H), of EGFP-Rab7a(Q67L) and mCherry-D4H (I), or of mCherry-D4H and filipin (J) in images similar to those in (G) (*n* = 24 and 32 cells for siControl and siPDZD8, respectively). (K) Confocal fluorescence images of HeLa cells transfected with siControl or siPDZD8 as well as with an expression vector for mCherry-D4H (red) and then metabolically labeled with filipin (shown blue in merged images). The boxed regions in the left panels are shown enlarged in those to the right. (L) Proportion of cells transfected as in (K) that showed an abnormal distribution of mCherry-D4H as indicated in Figure S5B (*n* = 229 cells in nine images and 207 cells in eight images for siControl and siPDZD8, respectively). All quantitative data are presented as box-and-whisker plots, with the boxes indicating the median and upper and lower quartile values and with the whiskers representing the maximum and minimum values. ∗∗p < 0.01, ∗∗∗p < 0.001 (Student’s t test). See also Figure S5.

To validate this model of lipid transfer by PDZD8, we next examined the subcellular distribution of PS and cholesterol in HeLa cells depleted of PDZD8 by transfection with a small interfering RNA (siRNA). The PS probes Lactadherin-C2 and Evectin2-PH have different targeting properties, detecting PS predominantly in the plasma membrane and in endosomes, respectively.^41^^,^^42^ We, therefore, examined PS dynamics between ER and Rab7-positive organelles with Evectin2-PH. We found that overlap of the ER marker mCherry-KDEL and green fluorescent protein (GFP)-tagged Evectin2-PH was lower in PDZD8-depleted cells than in control cells (Figures 5A and 5B). In addition, the overlap of mCherry-Rab7a(Q67L) and GFP-Evectin2-PH was higher in PDZD8-depleted cells than in control cells (Figures 5C and 5D). These results were thus consistent with the notion that PDZD8 transfers PS from Rab7-positive organelles to ER.

We next examined the localization of enhanced GFP (EGFP)-tagged KDEL and the cholesterol probe filipin in cells transfected with control or PDZD8 siRNAs. Overlap of EGFP-KDEL and filipin was higher in the PDZD8-depleted cells than in the control cells (Figures 5E and 5F). The cholesterol probe D4H can detect both cholesterol and its derivatives,^43^^,^^44^^,^^45^ and we detected mCherry-D4H signals within endolysosomes of HeLa cells in the steady state (Figure S5A). The localization of EGFP-Rab7a(Q67L), filipin, and mCherry-D4H overlapped considerably in control cells but not in PDZD8-depleted cells (Figure 5G). Statistical analysis with Pearson’s correlation coefficient showed that the overlap of Rab7a(Q67L) with either filipin or D4H was significantly lower in PDZD8-depleted cells than in control cells (Figures 5H and 5I). These results were thus consistent with the notion that PDZD8 transfers cholesterol from ER to Rab7-positive organelles. Together, our data thus provided support for the model proposed in Figure 4I, showing that PDZD8 promotes the exchange of PS and cholesterol between ER and Rab7-positive organelles. However, given that it is possible that PDZD8 transfers lipids in both directions between ER and Rab7-positive organelles, the amounts of lipids we detected in organelle membranes might reflect the net outcome of bidirectional transfer. In other words, at ER-endosome MCSs, at which PDZD8 is localized, cholesterol transport from ER to the Rab7-positive organelles may be preferentially enhanced.

Localization of the cholesterol probes filipin and mCherry-D4H overlapped substantially in control cells but hardly at all in PDZD8-depleted cells (Figures 5G, 5J, and 5K). Whereas mCherry-D4H showed an endolysosome-like distribution in control cells, it showed an ectopic distribution in the plasma membrane and intracellular fibrous structures as well as localization to endolysosome-like organelles in PDZD8-depleted cells (Figures 5K, 5L, and S5B). In addition, mCherry-D4H and EGFP-KDEL showed little overlap in either control or PDZD8-depleted cells (Figure S5C). These results suggested that PDZD8 transfers cholesterol from ER to Rab7-positive organelles and that such transfer might in turn promote the incorporation of CEs into endolysosomes.

### PDZD8 promotes fusion of Rab7-positive organelles and D4H-positive organelles

**Figure 6:**
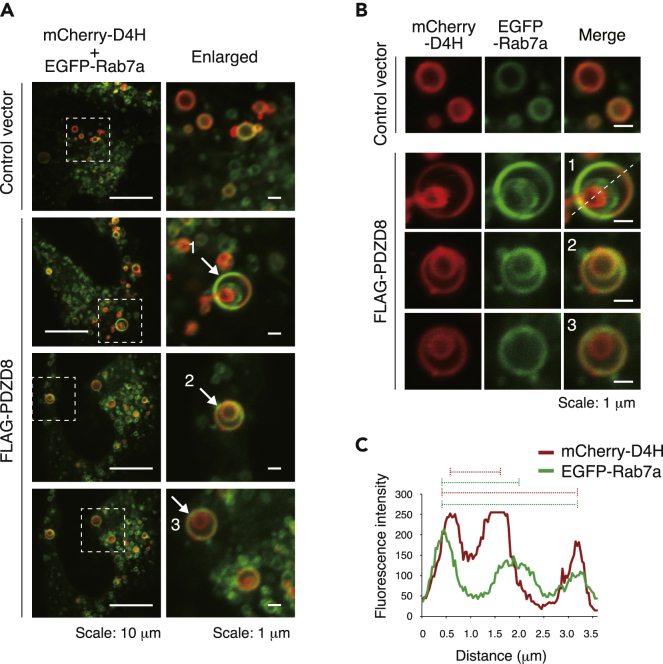
PDZD8 promotes fusion between D4H-positive and Rab7-positive organelles (A) Confocal fluorescence microscopy images of HeLa cells transfected with expression vectors for mCherry-D4H (red) and EGFP-Rab7a (green) as well as with either an expression vector for FLAG epitope-tagged mouse PDZD8(WT) or the corresponding control vector. The boxed regions in the left panels are shown enlarged in the right panels. (B) Double- or triple-layered organelles indicated by the arrows in (A) are shown at higher magnification. (C) Fluorescence intensity of mCherry-D4H (red) and EGFP-Rab7a (green) for structure 1 in (B). The bars above the graph indicate the distances between the corresponding peaks of fluorescence intensity.

Cholesterol promotes membrane fusion between Rab7-positive organelles (endolysosomes) and autophagosomes.^33^ Furthermore, active Rab7 facilitates the incorporation of LDs into lysosomes by lipophagy.^34^ PDZD8, which binds to active Rab7, might therefore play a role in cholesterol-dependent membrane fusion of Rab7-positive organelles and in lipophagy. Overexpression of PDZD8 in HeLa cells resulted in the occasional appearance of abnormal organelles consisting of double- or triple-layered structures with membranes positive for mCherry-D4H, EGFP-Rab7a, or both markers (Figures 6A and 6B). The outermost layer of the multilayered organelles was positive for both mCherry-D4H and EGFP-Rab7a and appeared to surround a D4H-positive organelle, consistent with the presence of an LD within a lysosome (Figure 6C). These abnormal structures, which were detected only in PDZD8-overexpressing cells, might result from a delay in the degradation of excessively incorporated LDs within lysosomes, with such excessive incorporation possibly being caused by the presence of an excessive amount of cholesterol in the lysosome membrane. These results thus indicated that PDZD8 facilitates the incorporation of LDs by and their fusion with lysosomes—that is, PDZD8 promotes LD degradation by lipophagy.

### PDZD8 regulates lipophagy by promoting lysosome maturation and fusion

**Figure 7:**
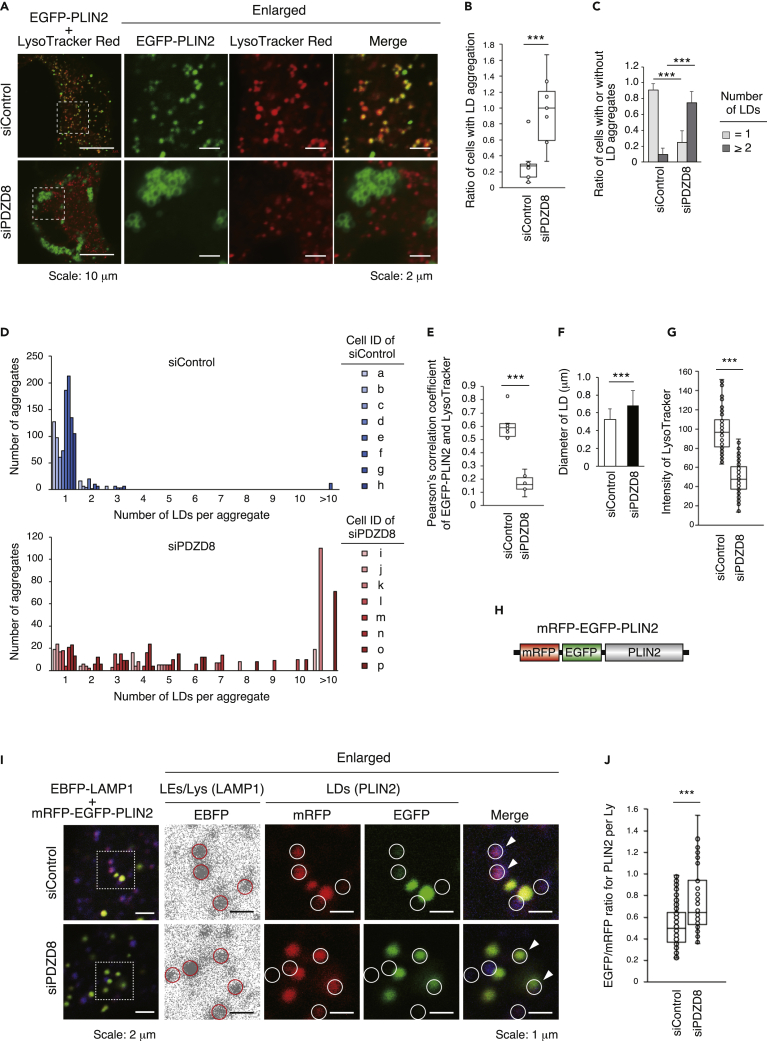
PDZD8 promotes lipophagy (A) Confocal fluorescence microscopy images of PC12 cells transfected with siControl or siPDZD8 as well as with an expression vector for EGFP-PLIN2 (green) and then metabolically labeled with LysoTracker Red (red). The boxed regions in the left panels are shown enlarged in those to the right. (B) Proportion of cells showing LD aggregation in images similar to those in (A) (*n* = 175 in 11 images and 140 cells in 10 images for siControl and siPDZD8, respectively). (C) Proportion of cells examined in (D) with LD aggregates containing ≥2 LDs. (D) Number of LDs per aggregate for eight individual cells determined from images similar to those in (A). (E) Pearson’s correlation coefficient for the colocalization of EGFP-PLIN2 and LysoTracker Red in images similar to those in (A) for the cells examined in (D). (F) Diameter of LDs determined for the cells examined in (D) (*n* = 1097 and 665 LDs, respectively). (G) Fluorescence intensity of LysoTracker Red per cell area in PC12 cells transfected with siControl or siPDZD8 as in Figure S6B (*n* = 93 or 99 lysosomes for siControl and siPDZD8, respectively). (H) Schematic representation of the structure of mRFP-EGFP-PLIN2. (I) Confocal fluorescence microscopy of PC12 cells transfected with siControl or siPDZD8 as well as with expression vectors for EBFP-LAMP1 (blue) and mRFP-EGFP-PLIN2 (red and green). The boxed regions in the left panels are shown at higher magnification than those to the right, with circles indicating LAMP1-positive lysosomes. Arrowheads indicate LDs enclosed within lysosomes. (J) Ratio of EGFP/mRFP fluorescence intensity for tagged PLIN2 within lysosomes as shown as in (I) (*n* = 108 or 94 lysosomes for siControl and siPDZD8, respectively). Quantitative data are presented as box-and-whisker plots or as means + SD. ∗∗∗p < 0.001 (Student’s t test). See also Figure S6.

We then examined lipophagy activity with the use of EGFP-tagged perilipin 2 (PLIN2), a marker of LDs. PC12 rat pheochromocytoma cells were transfected with control or PDZD8 siRNAs as well as with an expression vector for EGFP-PLIN2 and were also labeled with the lysosome marker LysoTracker Red. Fluorescence microscopic analysis revealed a significant aggregation of LDs in the PDZD8-depleted cells (Figures 7A, 7B, and S6A). Quantitative analysis revealed that, whereas most control cells did not contain LD aggregates, LDs in ∼70% of PDZD8-depleted cells were present in aggregates of two or more droplets (Figure 7C). The number of LDs per aggregate in individual PDZD8-depleted cells varied widely, with some aggregates containing >10 LDs (Figure 7D). The overlap of EGFP-PLIN2 and LysoTracker Red was also significantly reduced in PDZD8-depleted cells compared with control cells (Figure 7E). Furthermore, the size of LDs was significantly greater in PDZD8-depleted cells than in control cells (Figure 7F).

Endosomal maturation involves the conversion of LEs to lysosomes as well as lysosomal activation.^12^^,^^30^^,^^32^ Lysosome activity is dependent on a reduction in internal pH and can be measured with LysoTracker Red, which fluoresces maximally at low pH (∼pH 4–5). Lysosome activity was markedly reduced in PDZD8-depleted PC12 cells compared with control cells (Figures 7A, 7G, and S6B), consistent with PDZD8 promoting endosome maturation.^16^ We then examined lysosomal activity during lipophagy with the use of monomeric red fluorescent protein (mRFP)-EGFP-PLIN2, with the fluorescence of mRFP being stable and that of EGFP being reduced on exposure to the low pH of the lysosome lumen.^46^ The EGFP/mRFP fluorescence intensity ratio for tagged PLIN2 within lysosomes is thus inversely correlated with the activity of lysosomes and lipophagy (Figure 7H). PC12 cells transfected with control or PDZD8 siRNAs as well as with expression vectors for an enhanced blue fluorescent protein (EBFP)-tagged form of the lysosome marker protein LAMP1 and for mRFP-EGFP-PLIN2 were analyzed for the EGFP/mRFP ratio per EBFP-positive organelle. The ratio was found to be significantly increased in PDZD8-depleted PC12 cells compared with control cells (Figures 7I and 7J). These results thus suggested that PDZD8 contributes to the progression of lipophagy by promoting lysosome maturation.

## Discussion

We have here revealed the abnormal accumulation of CEs in the brain of PDZD8-KO mice and characterized its underlying mechanism. PDZD8 transfers cholesterol from ER to LEs/Lys and thereby promotes endosome maturation, leading to lysosome maturation and fusion with LDs and consequent CE degradation by lipophagy. CE accumulation in the PDZD8-KO brain is therefore the result of defective CE degradation as a consequence of impaired lipophagy (Figure S7). Our study thus provides insight into how the lipid transfer activity of PDZD8 might contribute to brain function and indicates that cholesterol storage disease is due to a defect in lipophagy.

Although our previous study suggested that PDZD8 possesses cholesterol extraction activity,^16^ with an improved liposome-FRET assay we now show that PDZD8 actually mediates PS and cholesterol transfer, including both extraction and insertion—that is, it possesses PS and cholesterol exchange transfer activity. However, NBD is a bulky tag and NBD-cholesterol may possible to change the physical property of cholesterol. Our present results then could be further confirmed by lipid transfer assays using dehydroergosterol (DHE), a naturally occurring fluorescent sterol analog.^47^ Intracellular cholesterol is transferred between several organelles including between ER and LEs/Lys as well as between ER and the plasma membrane through the action of various LTPs. In addition to PDZD8, LTPs such as NPC1/2, ORP1L, and STARD3 transfer cholesterol between ER and LEs/Lys.^48^^,^^49^^,^^50^ How these proteins might share the task of cholesterol transfer remains unknown, but PDZD8 and ORP1L are similar in that they both bind to Rab7 as well as to VAP.

LDs consist of a core of lipid esters surrounded by a single layer of phospholipids and cholesterol at the surface.^51^ The lipid esters in adipocytes consist mostly of TG, whereas those in nonadipocytes comprise mostly CEs.^52^ We found that only CEs manifest abnormal accumulation in the PDZD8-deficient brain, with the amounts of other lipids such as TG and FAs being unaffected. Specific accumulation of CEs has also been detected in induced pluripotent stem cell-derived neurons from individuals with Alzheimer’s disease, in Trem2- or ApoE-deficient glia, and in the brain of individuals with Huntington’s disease^3^^,^^5^^,^^53^ Furthermore, the accumulation of CEs in Trem2-or ApoE-deficient glial cells was enhanced by clozapine, a selective inhibitor of the mesolimbic dopaminergic system.^4^^,^^5^ Such association between specific accumulation of CEs in the central nervous system and neurological disease suggests that CEs must be maintained at low levels to ensure normal brain function. However, the reason for the accumulation of only CE but not of other lipids such as TG in PDZD8-deficient brain is unclear at this time.

We have demonstrated that lipophagy was impaired in the cholinergic neurons of the MHb in PDZD8-deficient mouse brain (Figures 3G–3J) and in the cultured dopaminergic neurons of PDZD8-deficient PC12 cells (Figure 7). On the other hand, we do not rule out the possibility of CE accumulation in PDZD8-deficient glial cells (astrocytes, microglia, and oligodendrocytes), which we have not obtained evidence to support such a possibility.

### Limitations of the study

This study demonstrates for the first time that PDZD8 plays a role in promoting lipophagy and that deficiency of PDZD8 leads to dyslipidemia in the mammalian brain. However, these evidences were limited to neuronal cell lines and mouse brains, and further confirmation of findings in humans is needed. Increased inflammation due to lipid accumulation in the brain has been suggested as a causative mechanism of neurological diseases in humans. Mutations in human PDZD8 have also been reported to be associated with abnormal brain development and intellectual disability. PDZD8 may therefore be a key molecule in elucidating the relationship between dyslipidemia and inflammation-dependent neurological disorders in the brain.

## STAR★Methods

### Key resources table

**Table:** 

REAGENT or RESOURCE	SOURCE	IDENTIFIER
**Antibodies**		

Anti-PDZD8 antibody produced in rabbit	Sigma-Aldrich	Cat#HPA015716; RRID:AB_1855162
Anti-Choline Acetyltransferase Antibody	Millipore	Cat#AB144P; RRID:AB_2079751
Alexa Fluor 488 goat anti-rabbit IgG	Invitrogen	Cat#A32731; RRID:AB_2633280
Alexa Fluor 555 donkey anti-goat IgG	Invitrogen	Cat# A21432; RRID:AB_2535853

**Bacterial and virus strains**		

*E*. *coli* DH5α	Takara	Cat#9057
BL21(DE3)pLysS	Thermo Fisher Scientific	Cat# C602003

**Biological samples**		

Mouse tissues	This paper	N/A

**Chemicals, peptides, and recombinant proteins**		

Hoechst 33342	Sigma-Aldrich	Cat#B2261
LysoTracker Red DND-99	Thermo Fisher Scientific	Cat#L7528
Filipin complex from *Streptomyces filipinensis*	Sigma-Aldrich	Cat#F9765
Digitonin	Tokyo Chemical Industry	Cat#D0540
Triton X-100	Nacalai Tesque	Cat#12969–25
Phosphate-buffered saline (PBS)	Wako	Cat#164–23551
Dulbecco’s modified Eagle’s medium (DMEM)	Wako	Cat#048–29763
RPMI-1640 Medium	Thermo Fisher Scientific	Cat#R8758
Fetal bovine serum (FBS)	Nichirei	Cat#175012
Horse serum	Thermo Fisher Scientific	Cat# 1998122
Nerve growth factor (NGF)	Merck Millipore	Cat#NC010
Poly-L-lysine (150–300 kDa)	Sigma-Aldrich	Cat#P4832
Trypsin	Wako	Cat#209–16941
X-tremeGENE 9 DNA Transfection Reagent	Roche	Cat#6365809001
Amaxa Cell Line Nucleofector® Kit V	Lonza	Cat#VCA-1003
Paraformaldehyde	Wako	Cat#162–16065
Surgipath FSC22	Leica	Cat#FSC22
Glutaraldehyde	Nisshin EM	Cat#3055–1
TAAB DMP-30	Nisshin EM	Cat#348
Isopropyl b-D-1-thiogalatopyranoside (IPTG)	Wako	Cat#092–05863
Ni-NTA agarose	Wako	Cat#141–09764
Imidazole	Wako	Cat#099–00013
POPC (16:0–18:1)	Avanti	Cat#850457C
POPE (16:0–18:1)	Avanti	Cat#850757C
POPS (16:0–18:1)	Avanti	Cat#840034C
Rhodamine-DPPE (18:1 Liss Rhod-PE)	Avanti	Cat#810150C
NBD-PS (16:0–12:0)	Avanti	Cat#810193C
NBD-PA (16:0–12:0)	Avanti	Cat#810174C
NBD-PC (16:0–12:0)	Avanti	Cat#810133C
NBD-PE (18:1–12:0)	Avanti	Cat#810156C
25-NBD Cholesterol	Avanti	Cat#810250C
DGS-NTA(Ni)	Avanti	Cat#790404C
Brain PI(4)P	Sigma-Aldrich	Cat#840045X
Recombinant His_6_-PDZD8 mutant proteins	This paper	N/A
Recombinant His_6_-GST protein	This paper	N/A
Rodent Diet CE-2	CLEA Japan	Cat#CE-2
High Fat Diet-32	CLEA Japan	Cat#HFD-32
RNeasy Lipid Tissue Mini Kit	QIAGEN	Cat#74804
ReverTra Ace® qPCR RT Master Mix with gDNA Remover	TOYOBO	Cat# FSQ-301
Thunderbird Next SYBR qPCR Mix	TOYOBO	Cat#QPX-201
MightyAmp DNA polymerase	Takara	Cat#R071A
PrimeSTAR HS DNA polymerase	Takara	Cat#R010A
Lipofectamine™ RNAiMAX Transfection Reagent	Promega	Cat#13778–150

**Experimental models: Cell lines**		

Human: HeLa	ATCC	Cat#CCL-2
Rat: PC12	Hokkaido Univ.	N/A

**Experimental models: Organisms/strains**		

C57BL/6 mice	SLC	C57BL/6
PDZD8-KO mice (C57BL/6 background)	This paper	RIKEN BRC (RBRC10434)

**Oligonucleotides**		

See Table S1 for primers	This paper	N/A
See Table S2 for siRNAs	This paper	N/A

**Recombinant DNA**		

pET-30	Novagen	Cat#69909
pcDNA3.1	Invitrogen	Cat#V79020
pEGFP	Clontech	Cat#6084–1
pmCherrry	Clontech	Cat#632524

**Software and algorithms**		

ZEN Microscopy Software	Zeiss	ZEN
BellCurve for Excel (statistical software)	Social Survey Research Information Co., Ltd.	BellCurve for Excel

**Other**		

Amicon Ultra-4	Merck Millipore	Cat#UFC800324
Mini Dialysis Kit	GE Healthcare	Cat#80648394
Mini-Extruder Heating Block	Avanti	Cat# 610024
Mini-Extruder Syringe	Avanti	Cat#610017
Sonicator Q55	QSonica	Cat#Q55
Spectrofluorometer	JASCO	Cat#FP8300
LSM800 confocal microscope with Airyscan	Zeiss	LSM800
JEM-1400 Plus instrument	JEOL	JEM-1400 Plus
Cryostat microtome (CM1850 UV)	Leica	CM1850 UV
StepOnePlus Real time PCR system	Applied Biosystems	StepOnePlus

### Resource availability

#### Lead contact

Further information and requests for resources and reagents should be directed to and will be fulfilled by the lead contact, Michiko Shirane (shiram@phar.nagoya-cu.ac.jp).

#### Materials availability

This study did not generate new unique reagents.

### Experimental model and subject details

#### Mutant mice

Generation of PDZD8-KO mice was described previously.^16^ In brief, Cas9 nickase mRNA (IDT 1074181, Alt-R S.p. Cas9 Nuclease 3NLS), Generic tracrRNA (IDT 1072532, Alt-R CRISPR-Cas9 tracrRNA), and target-specific crRNA (IDT, Custom Alt-R CRISPR crRNA) were mixed and injected into C57BL/6J mouse zygotes with the use of a Super Electroporator NEPA21 Type II (Nepagene). The 5′ and -3′ single guide RNAs with 20-nucleotide sequences targeted to exon 1 (which contains the start codon) of the PDZD8 gene (GenBank accession number NM_001033222) and containing protospacer-adjacent motif sequences were 5′-GCAGGCCGAGGGTTGCGGCGGGG-3′ and 5′-GCAGATTCCCAGCACGACCCTGG-3′, respectively. Potential predicted off-target sites were checked for at http://crispr.mit.edu. The resultant mutant mice were backcrossed with C57BL/6J mice before experiments. Animals were genotyped by PCR analysis with MightyAmp DNA polymerase (Takara). All mouse experiments were approved by the animal ethics committee of Nagoya City University. Primer sequences for genotyping PCR are provided in Table S1.

#### Mouse diets

The ND (Rodent Diet CE-2) and HFD (High Fat Diet-32) were obtained from CLEA Japan. The HFD contains a crude fat content of ∼32% including powdered beef tallow and high oleic–type safflower oil. Mice were fed the HFD (or ND) for 1 month before analysis.

### Method details

#### Plasmids

Construction of vectors encoding mouse PDZD8 (GenBank accession number CH466585.1) and its mutants was described previously.^16^ PDZD8 cDNA was amplified from C57BL/6J mouse brain by PCR with PrimeSTAR HS DNA polymerase (Takara) and specific primers, and it was then subcloned into pET30 (Novagen) or pcDNA3.1 (Invitrogen–Life Technologies). Human cDNAs encoding Rab7a (GenBank accession number NM_004637), LAMP1 (GenBank accession number BC025335), CD63 (GenBank accession number NM_001780), and KDEL with the signal sequence of calreticulin (GenBank accession number NM_004343) were amplified from HeLa cells by PCR with specific primers and were then subcloned into pEGFP or pmCherrry (Clontech). Rab7a(Q67L) cDNA was generated by PCR-mediated mutagenesis of Rab7a. EBFP cDNA was generated by PCR-mediated mutagenesis of EGFP cDNA. The mCherry-D4H,^44^ mRFP-EGFP-PLIN2,^46^ and GFP-Evectin2-2×PH^54^ vectors were described previously.

#### Antibodies and reagents

Rabbit polyclonal antibodies to PDZD8 were from Sigma-Aldrich, and guinea pig polyclonal antibodies to AChT were from Sigma-Aldrich. Alexa Fluor 488 goat anti-rabbit IgG and Alexa Fluor 555 donkey anti-goat IgG as well as LysoTracker Red were from Thermo Fisher Scientific. Hoechst 33342 and filipin were from Sigma-Aldrich. Digitonin was from Tokyo Chemical Industry.

#### Cell culture and transfection

HeLa cells were cultured under a humidified atmosphere of 5% CO_2_ at 37°C in Dulbecco’s modified Eagle’s medium (DMEM, Wako) supplemented with 10% fetal bovine serum (FBS, Nichirei). They were transfected with expression vectors for 24 h with the use of the ExtremeGENE9 reagent (Roche). PC12 cells were cultured under a humidified atmosphere of 5% CO_2_ at 37°C in DMEM supplemented with 10% FBS and on plates coated with poly-L-lysine (150–300 kDa, Sigma) before exposure to mouse submaxillary gland nerve growth factor (Merck Millipore) at 100 ng/mL in RPMI 1640 supplemented with 1% horse serum (Thermo Fisher Scientific). They were transfected with siRNAs or expression vectors with the use of a Nucleofector system (Lonza).

#### RNA interference

Stealth siRNAs targeted to human or rat PDZD8 mRNA were obtained from Invitrogen–Life Technologies. The siRNAs were introduced into HeLa cells with the use of the Lipofectamine RNAiMAX reagent (Invitrogen–Life Technologies), and the cells were then cultured for 72 h before experiments. The siRNAs were introduced into PC12 cells with the use of a Nucleofector instrument (Lonza), and the cells were then cultured for 72 h before experiments. Results are shown for human and rat siRNAs #1, but similar findings were obtained with the other two siRNAs for each species. Stealth siRNA sequences are provided in Table S2.

#### RT and real-time PCR analysis

Total RNA was isolated from mouse brain with the use of an RNeasy Kit (Qiagen) and was subjected to RT with the use of ReverTra Ace qPCR RT Master Mix with gDNA Remover (Toyobo). The resulting cDNA was subjected to real-time PCR analysis with Thunderbird Next SYBR qPCR Mix (Toyobo) in a StepOnePlus Real-Time PCR System (Thermo Fisher Scientific). The amounts of target mRNAs were normalized by that of HPRT mRNA for relative quantification. Plasmid cDNA was used as a standard for absolute quantitative analysis. Primer sequences for PCR are provided in Table S1.

#### Fluorescence imaging of live cells

Cells that had been transfected with plasmids encoding fluorescently tagged proteins or metabolically labeled with fluorescent probes were observed with an LSM800 confocal microscope (Zeiss), and the images were processed for calculation of fluorescence intensity with ZEN imaging software (Zeiss). LysoTracker Red (0.5 μM) was added to cells for 1 h at 37°C. Filipin (5 μg/mL) was added to cells for 1 h at 37°C after permeabilization with digitonin (3 μg/mL) for 10 min at 37°C.

#### Immunohistofluorescence analysis

Mouse brain was fixed with 3.7% paraformaldehyde in phosphate-buffered saline (PBS, Wako), exposed consecutively to 15% and 30% sucrose in PBS for 2 days, and embedded in Surgipath FSC22 (Leica). Thin sections were prepared with a cryostat microtome (CM1850 UV, Leica) and stained consecutively with primary antibodies and Alexa Fluor–labeled secondary antibodies in PBS containing 0.5% Triton X-100. Nuclei were stained with Hoechst 33342 (Wako) as indicated. The sections were observed with an LSM800 confocal microscope (Zeiss).

#### TEM

Mouse brain was fixed with 2% glutaraldehyde (Nisshin EM) in 0.05 M cacodylate buffer containing 5 mM CaCl_2_. The fixed tissue was washed in cacodylate buffer with CaCl_2_, exposed to buffered 1% OsO_4_ plus 0.8% K_4_[Fe(CN)_6_]·3H_2_O for 1 to 2 h, dehydrated with acetone or ethanol, and embedded in Epon-Araldite or Spurr’s medium (Nisshin EM). Thin sections were stained with uranyl acetate and lead citrate and observed with a JEM-1400 Plus instrument (JEOL).

#### Expression and purification of recombinant proteins

Recombinant His_6_-tagged proteins were expressed in and purified from *Escherichia coli*. The BL21(DE3)pLys bacterial cells were transformed with pET30-based vectors, cultured, and then exposed to 0.5 mM isopropyl-β-D-thiogalactopyranoside for 16 h at 10°C. The cells were then subjected to ultrasonic treatment, and the soluble fraction of the cell lysates was isolated. The expressed His_6_-tagged proteins were purified with the use of Ni-NTA agarose (Wako) and were eluted with imidazole (Wako). The purified proteins were concentrated with an Amicon Ultra device (Merck Millipore) and dialyzed against PBS with the use of a Mini Dialysis Kit (GE Healthcare).

#### Lipid transfer (liposome-FRET) assay

The liposome-FRET assay was modified compared with that described previously.^16^ All lipids were obtained from Avanti Polar Lipids: POPC (16:0–18:1), 1-palmitoyl-2-oleoyl-*sn*-glycero-3-phosphocholine; POPE (16:0–18:1), 1-palmitoyl-2-oleoyl-*sn*-glycero-3-phosphoethanolamine; POPS (16:0–18:1), 1-palmitoyl-2-oleoyl-*sn*-glycero-3-phospho-L-serine; rhodamine-DPPE (18:1 Liss Rhod-PE), 1,2-dioleoyl-*sn*-glycero-3-phosphoethanolamine-*N*-(lissamine rhodamine B sulfonyl); NBD-PS (16:0–12:0), 1-palmitoyl-2-{12-[(7-nitro-2-1,3-benzoxadiazol-4-yl)amino]dodecanoyl}-*sn*-glycero-3-phosphoserine (ammonium salt); NBD-PE (18:1–12:0), 1-oleoyl-2-{12-[(7-nitro-2-1,3-benzoxadiazol-4-yl)amino]dodecanoyl}-*sn*-glycero-3-phosphoethanolamine; NBD-PC (16:0–12:0), 1-oleoyl-2-{12-[(7-nitro-2-1,3-benzoxadiazol-4-yl)amino]dodecanoyl}-*sn*-glycero-3-phosphocholine; NBD-PA (16:0–12:0), 1-palmitoyl-2-{12-[(7-nitro-2-1,3-benzoxadiazol-4-yl)amino]dodecanoyl}-*sn*-glycero-3-phosphate (ammonium salt); and 25-NBD-cholesterol, 25-{*N*-[(7-nitro-2-1,3-benzoxadiazol-4-yl)methyl]amino}-27-norcholesterol. Liposomes were prepared with the use of a Q55 Sonicator (QSonica) and Mini-Extruder (Avanti Polar Lipids). Donor liposomes for assay of phospholipid transfer [final concentration of 6.88 μM, containing 2.5 μM PC, 2.5 μM PE, 0.13 μM rhodamine-labeled PE, 0.5 μM NBD-labeled phospholipid, and 1.25 μM DGS-NTA(Ni)] or of cholesterol transfer [final concentration of 9.38 μM, containing 2.5 μM PC, 2.5 μM PE, 2.5 μM PS, 0.13 μM rhodamine-labeled PE, 0.5 μM NBD-labeled cholesterol, and 1.25 μM DGS-NTA(Ni)] and acceptor liposomes [final concentration of 25 or 25.5 μM, containing 15 μM PC and 10 μM PE, with or without 0.5 μM PI(4)P] were mixed, His_6_-tagged proteins were added to a final concentration of 10 nM, and the mixtures were incubated at 25°C. The fluorescence (*F*) of NBD was measured with a spectrofluorometer (FP8500, JASCO) for 1800 s at 5-s intervals, with excitation at 460 nm and emission at 535 nm. For measurement of maximal fluorescence (*F*_max_), Triton X-100 (final concentration of 0.05%) was added to the reaction mixture at 1800 s. The amount of transferred phospholipid or cholesterol was calculated as: {[*F*(His_6_-PDZD8) – *F*(His_6_-GST)]/*F*_max_} × 0.5 μM.

#### Lipidome analysis

Lipids were extracted from frozen brain or liver by the method of Bligh and Dyer,^55^ with some modifications. The amounts of FAs, cholesterol, and CEs in the brain and liver were quantified by supercritical fluid chromatography (SFC) with a C_18_ column coupled with a triple-quadrupole mass spectrometer (C_18_-SFC/MS/MS analysis).^56^ The levels of other lipids (PC, PE, PS, PG, PI, PA, LPC, LPE, MG, DG, TG, SM, Cer, and HexCer) were quantified by SFC with a diethylamine (DEA) column coupled with a triple-quadrupole mass spectrometer (DEA-SFC/MS/MS analysis).^57^ Details of sample preparation and conditions for the analysis of hydrophobic metabolites are described below. Optimized multiple reaction monitoring (MRM) parameters for lipids are provided in Table S3. Raw data for lipidome analysis are provided in Table S4. Source data for lipidome analysis, related to Figure 1B, Table S5. Source data for lipidome analysis, related to Figure 1C, Table S6. Source data for lipidome analysis, related to Figures 1D and 1E, Table S7. Lipid amount ratio (KO/WT), related to Figure 1B, Table S8. Lipid amount ratio (KO/WT), related to Figure 1C, Table S9. Lipid amount ratios (KO/WT and HFD/ND), related to Figures 1D and 1E.

#### Sample preparation for lipidomics analysis

Lipids were extracted from frozen brain (20–55 mg) or frozen liver (35–40 mg) with 960 μL of extraction solvent (methanol/chloroform/water, 5:2:2, v/v/v) supplemented with 20 μL of internal standard A (Mouse SPLASH Lipidomix Mass Spec Standard, Avanti Polar Lipids)—containing 2.0 nmol of PC 15:0–18:1 (d_7_), 0.14 nmol of PE 15:0–18:1 (d_7_), 0.40 nmol of PS 15:0–18:1 (d_7_), 0.10 nmol of PG 15:0–18:1 (d_7_), 0.40 nmol of PI 15:0–18:1 (d_7_), 0.20 nmol of PA 15:0–18:1 (d_7_), 0.90 nmol of LPC 18:1 (d_7_), 0.04 nmol of LPE 18:1 (d_7_), 5.0 nmol of CE 18:1 (d_7_), 0.30 nmol of DG 15:0–18:1 (d_7_), 0.70 nmol of TG 15:0–18:1 (d_7_)–15:0, and 0.40 nmol of SM d18:1–18:1 (d_9_)—and 20 μL of internal standard B (Avanti Polar Lipids) containing 2.0 nmol of MG 18:1 (d_7_), 0.04 nmol of Cer d18:1 (d_7_)–15:0, 0.10 nmol of HexCer d18:1 (d_7_)–18:1, and 6.0 nmol of cholesterol (d_7_). The samples were vigorously agitated with a vortex mixer for 1 min and subjected to ultrasonic treatment for 5 min. Proteins were then precipitated by incubation on ice for 5 min and separated by centrifugation at 16,000 × *g* for 5 min at 4°C. The resultant supernatant was collected and 700 μL were transferred to clean tubes, and the protein concentration of the pellet was determined with a Pierce BCA Protein Assay Kit (Thermo Fisher Scientific). The supernatant was mixed with 235 μL of chloroform and 155 μL of water, and the aqueous and organic layers were separated by vortex-mixing and subsequent centrifugation at 16,000 × *g* for 5 min at 4°C. The organic (lower) layer (250 μL) obtained by phase separation was dried under a stream of nitrogen and stored at −80°C until analysis. For analysis, the dried sample was reconstituted with 80 μL of methanol/chloroform (1:1, v/v). Lipidomics analysis was performed by C_18_-SFC/MS/MS and DEA-SFC/MS/MS.

#### C_18_-SFC/MS/MS analysis for determination of FAs, cholesterol, and CEs

The conditions for C_18_-SFC (Nexera UC system, Shimadzu) were as follows: column, ACQUITY UPC^2^ HSS C18 SB (3.0-mm inner diameter by 100 mm, 1.8-μm particle size; Waters); injection volume, 2 μL; column temperature, 50°C; mobile phase A, supercritical carbon dioxide; mobile phase B (modifier) and make-up pump solvent, methanol/water (95:5, v/v) with 0.1% (w/v) ammonium acetate; flow rate of mobile phase, 1.0 mL/min; flow rate of make-up pump solvent, 0.1 mL/min; and back pressure regulator, 10 MPa. The gradient conditions were as follows: 0–50% B, 0–25 min; 50% B, 25–28 min; and 0% B, 28–30 min. The analysis conditions for triple-quadrupole mass spectrometry (TQMS, LCMS-8060, Shimadzu) were as follows: polarity, positive and negative ionization; electrospray voltage, 4 kV in the positive ion mode and −3.5 kV in the negative ion mode; nebulizer gas flow rate, 3.0 L/min; drying gas flow rate, 10.0 L/min; desolvation line temperature, 250°C; heat block temperature, 400°C; and detector voltage, 2.16 kV. The MRM parameters per one time period were as follows: limit on number of MRM transitions, 150; dwell time, 2 ms; pause time, 2 ms; and polarity switching time, 5 ms. Optimized MRM parameters for FAs, cholesterol, and CEs are shown in Table S3.

#### DEA-SFC/MS/MS analysis for quantitative lipidomics

The gradient conditions for DEA-SFC with an ACQUITY UPC^2^ Torus DEA column (3.0-mm inner diameter by 100 mm, 1.7-μm particle size; Waters) were as follows: 1% B, 0–1 min; 1–75% B, 1–24 min; 75% B, 24–26 min; and 1% B, 26–30 min. Other optimized MRM parameters for lipids (PC, PE, PS, PG, PI, PA, LPC, LPE, MG, DG, TG, SM, Cer, and HexCer) are shown in Table S3. The other DEA-SFC and TQMS parameters were the same as those for C_18_-SFC/MS/MS analysis.

### Quantification and statistical analysis

Quantitative data are presented as means +SD where indicated and were analyzed with Student’s t test or Pearson’s correlation coefficient as performed with ZEN imaging software (Zeiss). A p value of <0.05 was considered statistically significant.

## Data Availability

All data reported in this paper will be shared by the lead contact upon request. This paper does not report original code. Any additional information required to reanalyze the data reported in this paper is available from the lead contact upon request.

## Supplemental information

Document S1. Figures S1–S7 and Tables S1 and S2

Table S3. Analytic conditions for lipidomics analysis by C18-SFC/MS/MS and DEA-SFC/MS/MS, related to Figure 1 and 2

Table S4. Source data for lipidome analysis, related to Figure 1B Mouse: 7 M, n = 3. Tissue: Brain-BG and Cx. Amount of lipid (pmol/mg protein).

Table S5. Source data for lipidome analysis, related to Figure 1C Mouse: 5 M, n = 2. Tissue: Brain-BG and liver. Amount of lipid (pmol/mg protein). ND in all samples are shown in red.

Table S6. Source data for lipidome analysis, related to Figures 1D and 1E Mouse: 3 M. Tissue: Brain-BG and liver. ND or HFD (for 1 M). Amount of lipid (pmol/mg protein). ND in all samples are shown in red.

Table S7. Lipid amount ratio (KO/WT), related to Figure 1B Mouse: 7 M, n = 3. Tissue: Brain-BG and Cx. Heat map of ratio of lipid (KO/WT).

Table S8. Lipid amount ratio (KO/WT), related to Figure 1C Mouse: 5 M, n = 2. Tissue: Brain-BG and liver. Heat map of ratio of lipid (KO/WT).

Table S9. Lipid amount ratios (KO/WT and HFD/ND), related to Figures 1D and 1E Mouse: 3 M. Tissue: Brain-BG and liver. ND or HFD (for 1 M). Heat map of ratio of lipid (KO/WT and HFD/ND).
