# Supplementary material for: PDZD8-deficient mice accumulate cholesteryl esters in the brain as a result of impaired lipophagy
Source: iScience. 2022 Nov 16;25(12):105612. doi: 10.1016/j.isci.2022.105612 (PMC9709239; doi:10.1016/j.isci.2022.105612)
Supplement: Document S1. Figures S1–S7 and Tables S1 and S2 [file mmc1.pdf]

iScience, Volume *25*

Supplemental information

PDZD8-deficient mice accumulate cholesteryl esters in the brain as a result of impaired lipophagy

Keiko Morita, Mariko Wada, Kohta Nakatani, Yuki Matsumoto, Nahoki Hayashi, Ikuko Yamahata, Kotone Mitsunari, Nagi Mukae, Masatomo Takahashi, Yoshihiro Izumi, Takeshi Bamba, and Michiko Shirane

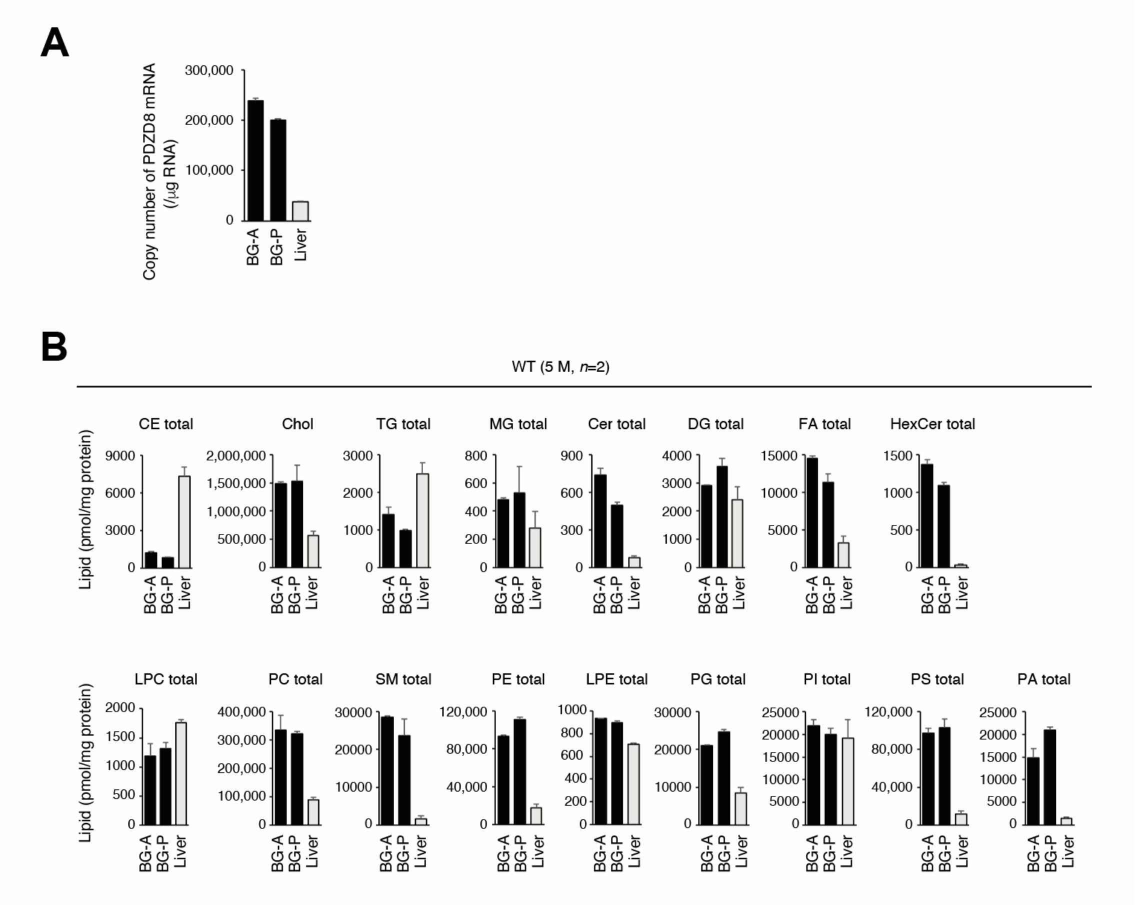

Figure S1. Comparison of the level of PDZD8 and CEs between the brain and the liver, related to Figure 1 and 2

(A) Copy number for PDZD8 mRNA per microgram of total RNA in the anterior (-A) and posterior (-P) portions of the BG and in the liver of WT mice at 3 months of age. Data are means + SD (*n* = 2 mice).

(B) Amounts of each lipid class in BG-A, BG-P, and liver of WT mice at 5 months of age (*n* = 2 mice). Data are means + SD.

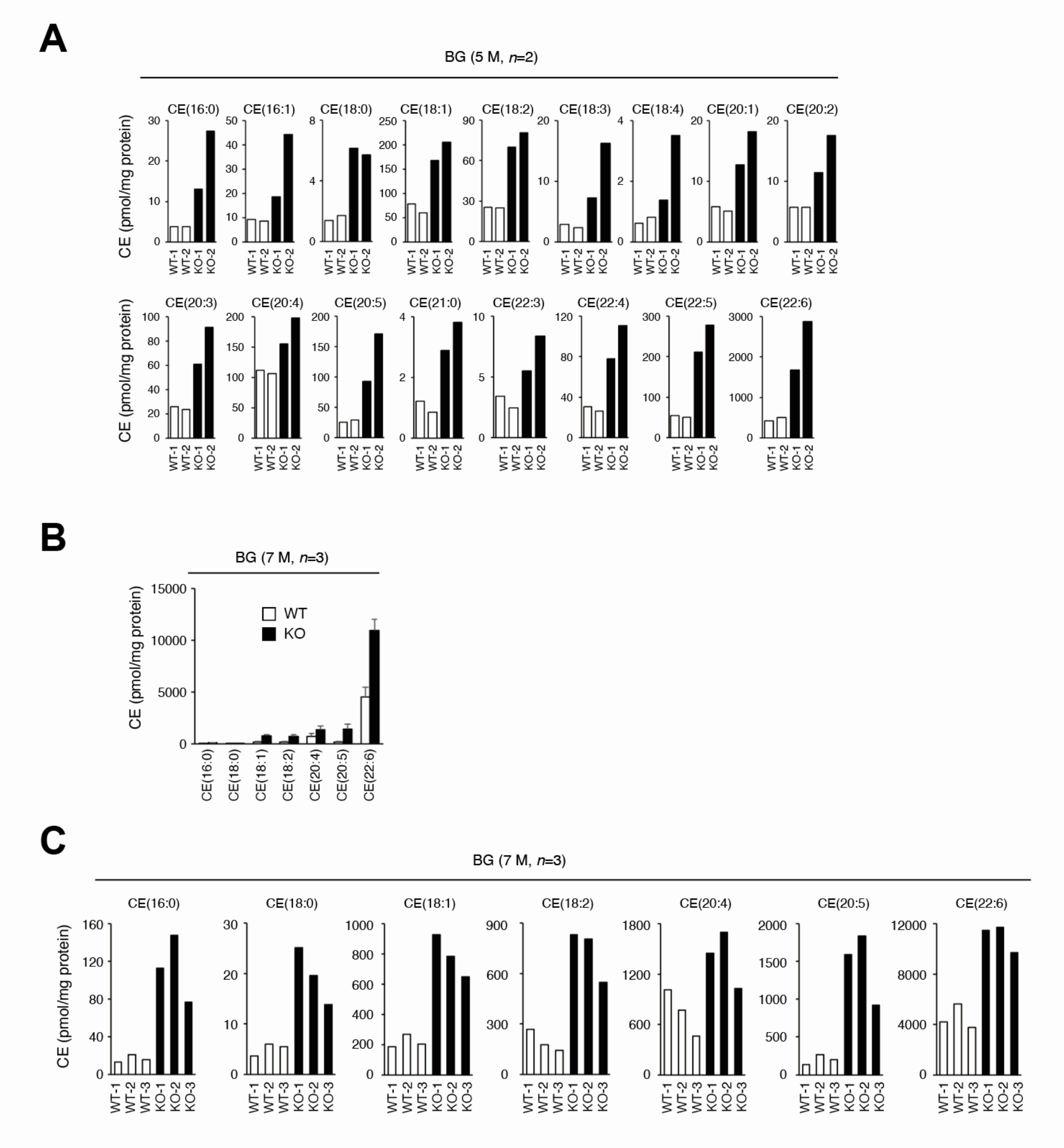

Figure S2. Abnormal CE accumulation in the brain of PDZD8-KO mice, related to Figure 1 and 2

(A) Amount of each type of CE in the BG for individual WT and PDZD8-KO mice as in Figure 1C.

(B) Amount of each type of CE in the BG for WT and PDZD8-KO mice as in Figure 1B. Data are means + SD.

(C) Amount of each type of CE in the BG for individual WT and PDZD8-KO mice as in Figure 1B.

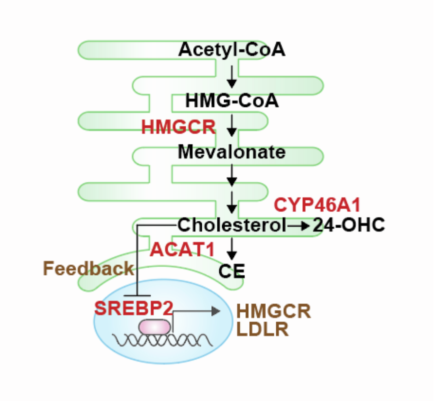

Figure S3. Schematic representation of cholesterol synthesis and metabolism in ER and its feedback regulation in the nucleus, related to Figure 3A

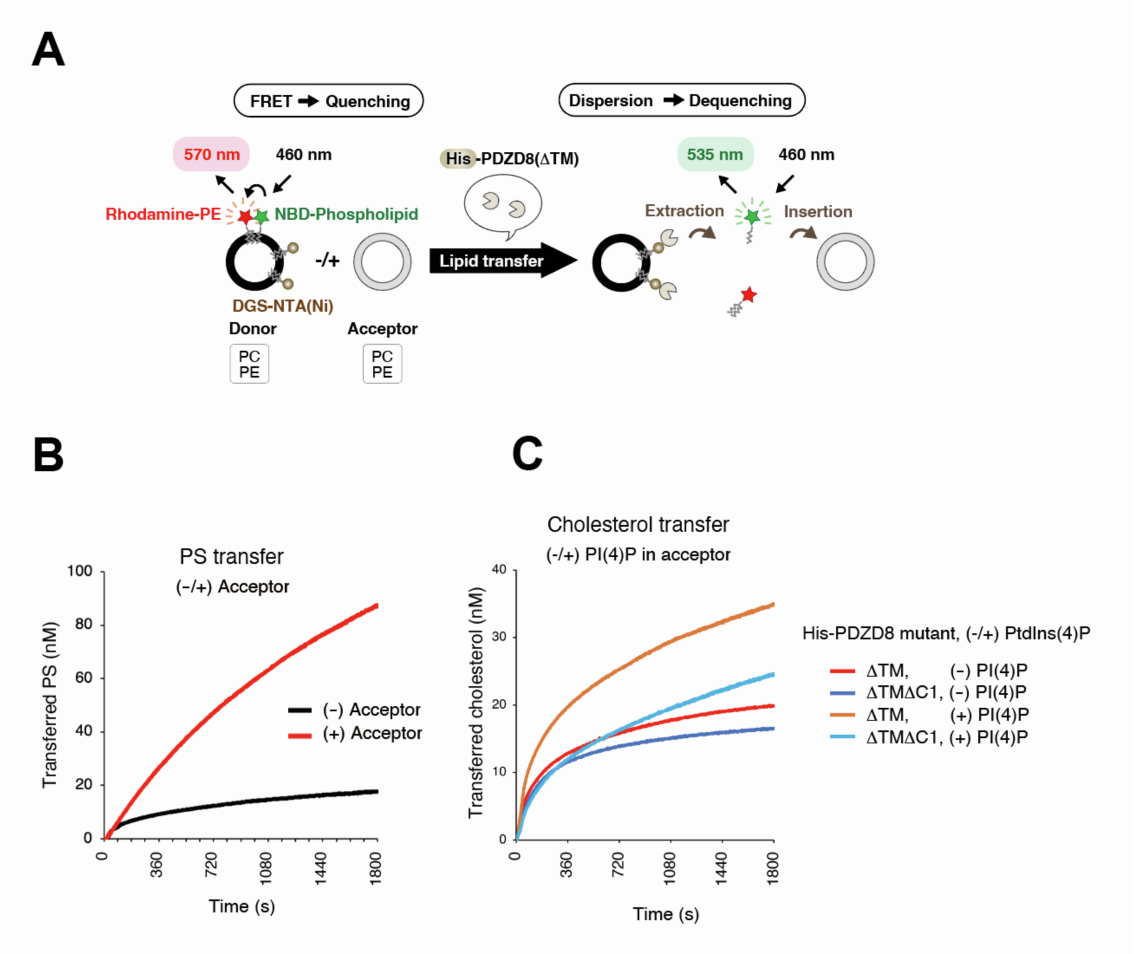

Figure S4. PS and cholesterol transfer activity of PDZD8, related to Figure 4

(A) Schematic representation of the liposome-FRET assay for phospholipid transfer by His_6-PDZD8(ΔTM) as performed with donor liposomes containing rhodamine-PE, NBD-phospholipid, and DGS-NTA(Ni) and in the absence or presence of acceptor liposomes. The lipid constituents of liposomes are shown in the boxes below.

(B) PS transfer activity of PDZD8(ΔTM) in the absence or presence of acceptor liposomes. The amount of transferred PS (nM) is shown.

(C) Cholesterol transfer activity of the indicated PDZD8 deletion mutants in the presence of acceptor liposomes containing or not containing PI(4)P.

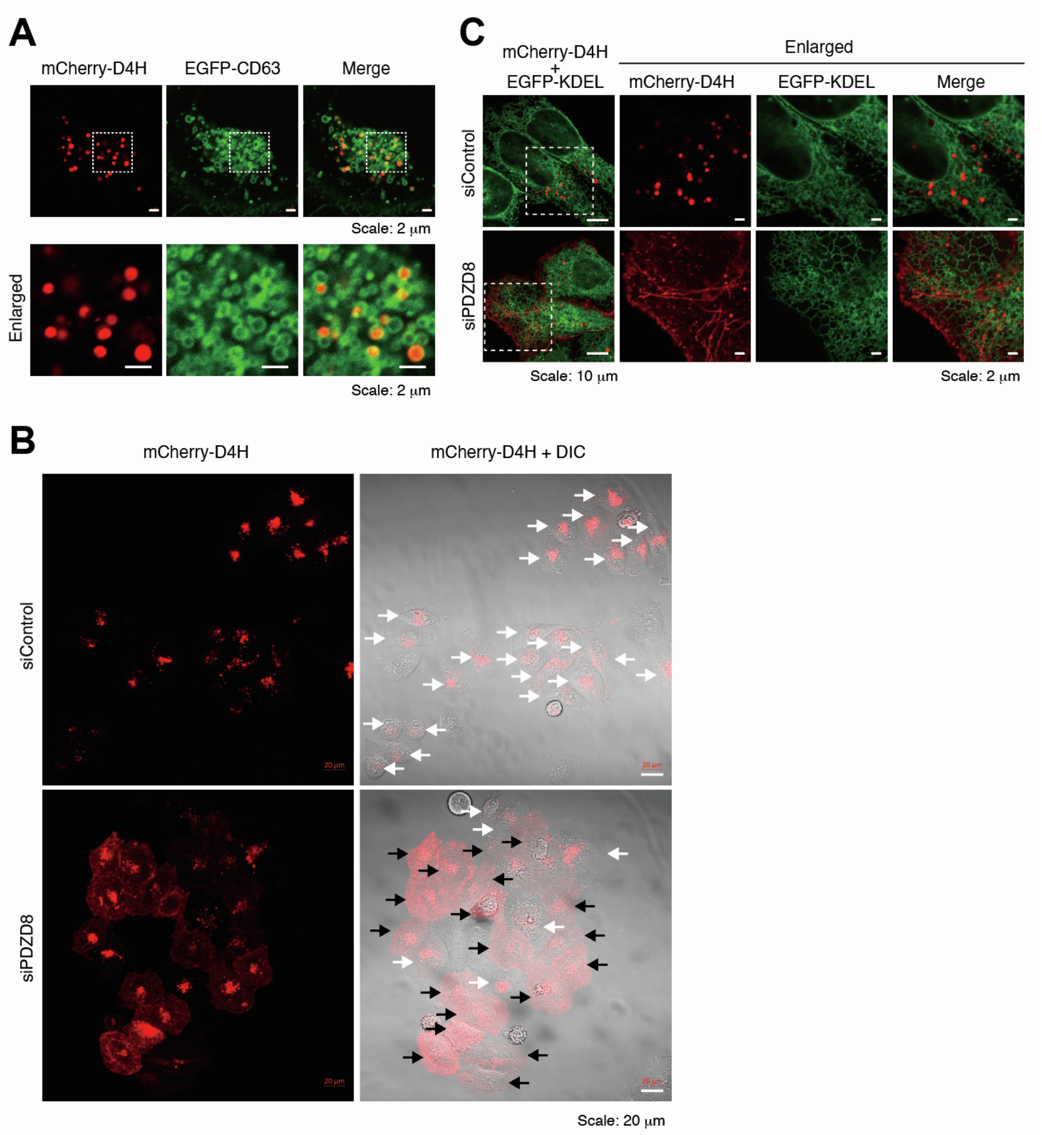

Figure S5. Abnormal distribution of D4H in PDZD8-depleted cells, related to Figure 5

(A) HeLa cells transfected with mCherry-D4H (red) and EGFP-CD63 (green). CD63 is a marker for endolysosomes.

(B) HeLa cells transfected with siControl or siPDZD8 as well as mCherry-D4H (red). Overlays of the fluorescence images with DIC images are also shown. White or black arrows indicate cells with normal or abnormal distribution of mCherry-D4H, respectively.

(C) HeLa cells transfected with siControl or siPDZD8 as well as with mCherry-D4H (red) and EGFP-KDEL (green).

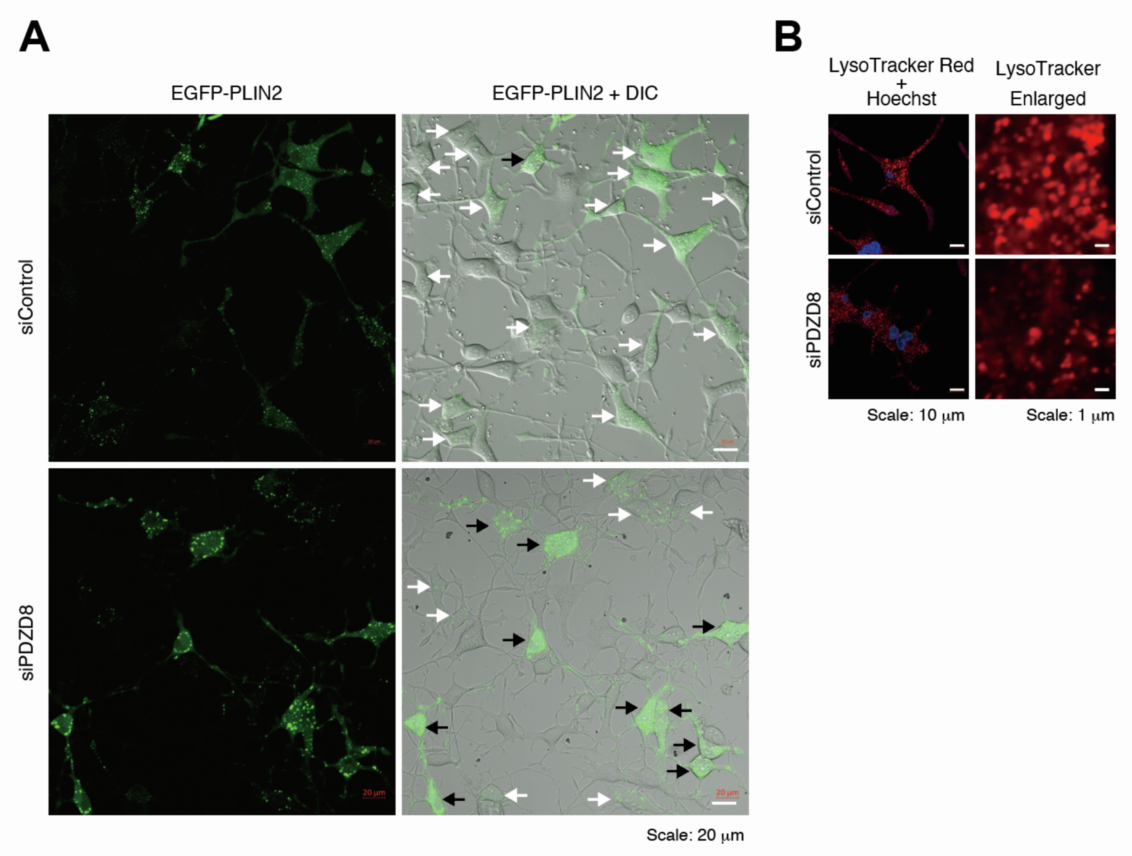

Figure S6. Abnormal aggregation of LDs and attenuated lysosome activity in PDZD8-depleted cells, related to Figure 7

(A) Confocal fluorescence microscopy of PC12 cells transfected with siControl or siPDZD8 as well as with an expression vector for EGFP-PLIN2 (green). Overlays of the fluorescence images with DIC images are also shown. White or black arrows indicate cells without or with LD aggregation, respectively.

(B) Confocal fluorescence microscopy images of PC12 cells transfected with siControl or siPDZD8 and then metabolically labeled with LysoTracker Red (red) and stained with Hoechst 33342 (blue). Enlarged images of LysoTracker Red are also shown in the right panels.

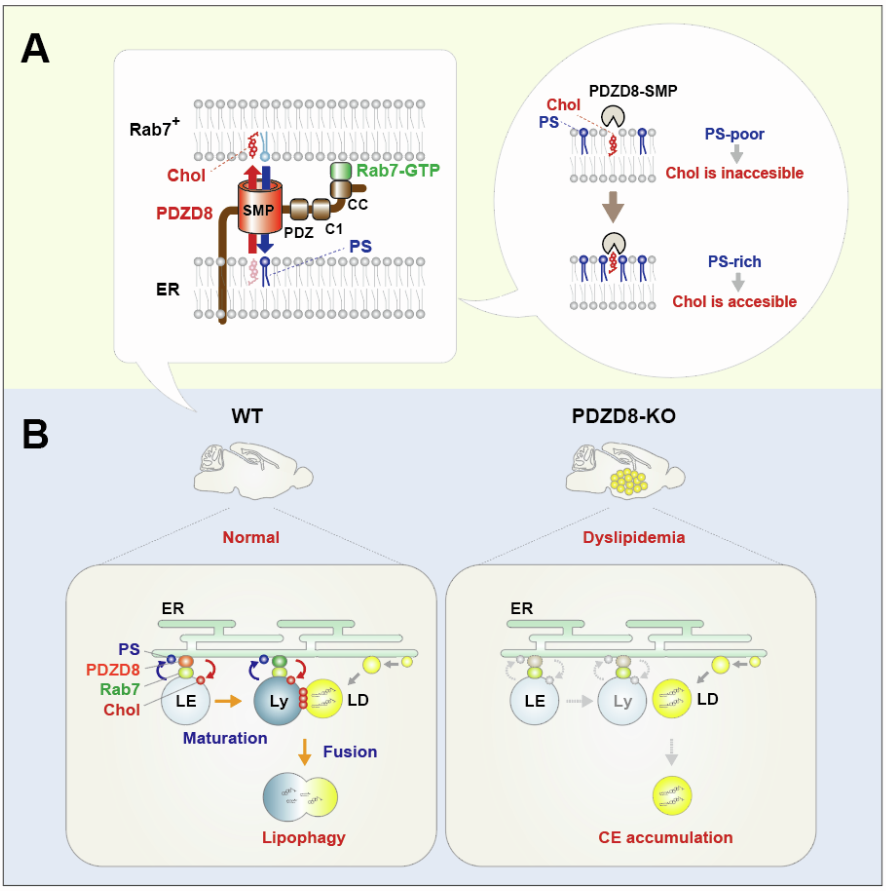

Figure S7. Mechanisms for lipid exchange by PDZD8 and for abnormal CE accumulation in the brain of PDZD8-deficient mice, related to Figure 1, 4, 7

(A) Mode of action for lipid exchange transfer by PDZD8. PDZD8 transfers PS from Rab7-positive organelles to ER as well as cholesterol from ER to Rab7-positive organelles in a manner dependent on its SMP domain (left). The SMP domain is able to access cholesterol only in PS-rich domains of a lipid bilayer (right).

(B) CEs are degraded normally in the WT brain, whereas they accumulate as a result of impaired lipophagy in the PDZD8-KO brain. PDZD8 transfers cholesterol from ER to Rab7-positive organelles such as LEs/Lys and thereby promotes lysosome maturation. The high concentration of cholesterol in the lysosome membrane is indispensable for the fusion of these organelles with LDs during lipophagy (left). Deficiency of PDZD8 results in insufficient maturation and fusion of lysosomes, leading to CE accumulation and consequent dyslipidemia in the brain (right).

Table S1 | Primer sequences for PCR, related to STAR METHODS.

PDZD8-KO mouse genotyping	Sequence
#1	5'-CTCAACAGACCCAGGAGAGG-3'
#2	5'-AGCCCGACTTATCCAGGTCT-3'
#3	5'-CGCTGGAGACCTGCTACTTC-3'

Mouse HPRT	Sequence
Forward	5'-TGCTCGAGATGTCATGAAGG-3'
Reverse	5'-TATGTCCCCCGTTGACTGAT-3'

Mouse HPRT	Sequence
Forward	5'-TGCTCGAGATGTCATGAAGG-3'
Reverse	5'-TATGTCCCCCGTTGACTGAT-3'

Mouse PDZD8	Sequence
Forward	5'-TCAACTGATGGGTATGCTGG-3'
Reverse	5'-ATAGCAATGAGCCGATCTCC-3'

Mouse Drd2	Sequence
Forward	5'-ATCTTGAACCTGTGTGCCATC-3'
Reverse	5'-GTTGAGTCCAAAGAGCAGTGG-3'

Mouse DAT	Sequence
Forward	5'-CAACGGTGGCATCTATGTCTT-3'
Reverse	5'-TGACCACGACCACATACAGAA-3'

Mouse NET	Sequence
Forward	5'-TGTGTTGTCACCATCAGCAC-3'
Reverse	5'-AAGCACCGCAAACAGAATGG-3'

Mouse BDNF	Sequence
Forward	5'-CGCAAACATGTCTATGAGGGT-3'
Reverse	5'-TTGGATACCGGGACTTTCTCT-3'

Mouse SREBP2	Sequence
Forward	5'-GCGTTCTGGAGACCATGGA-3'
Reverse	5'-ACAAAGTTGCTCTGAAAACAAATCA-3'

Mouse HMGCR	Sequence
Forward	5'-ATTCCAGCCAAGGTGGTGAGAG-3'
Reverse	5'-GCCACATGCGATGTAGATAGCAG-3'

Mouse LDLR	Sequence
Forward	5'-TCTTCCCTATTGCACTGGTT-3'
Reverse	5'-ATCTGTCTTGAGGGGTAGGT-3'

Mouse ACAT1	Sequence
Forward	5'-CCTCCCGGTTCATTCTGATA-3'
Reverse	5'-GCTTGATTTCTCCTTGGCTG-3'

Mouse CYP46A1	Sequence
Forward	5'-TCCTCAAAGCTGAAGAGGGA-3'
Reverse	5'-CTTGGAACCGACAACCTCAT-3'

Table S2 l Stealth siRNA sequences, related to STAR METHODS.

Human PDZD8	Sequence
#1	5'-GGAGUUCUAUUAAGACGGUUGAAUU-3'
#2	5'-CCAUUUGGUUGAAGAAGUUUCUGUU-3'
#3	5'-UGCAGUUAAAGAAAUUGGUCGUGAU-3'

Rat PDZD8	Sequence
#1	5'-GGGCCGGCTTAAAGTTACATTGCTA-3'
#2	5'-CAGTCCCAAACGTACTCCAACAACA-3'
#3	5'-GAGGTGGCTTTAGGATGCCTAGCTA-3'
